# Facilitators and barriers influencing weight management behaviours during pregnancy: a meta-synthesis of qualitative research

**DOI:** 10.1186/s12884-022-04929-z

**Published:** 2022-09-05

**Authors:** Tamara Escañuela Sánchez, Sarah Meaney, Caroline O’Connor, Laura Linehan, Keelin O’Donoghue, Molly Byrne, Karen Matvienko-Sikar

**Affiliations:** 1 Department of Obstetrics and Gynaecology, Pregnancy Loss Research Group, University College Cork. Cork University Maternity Hospital, Cork, Ireland; 2 INFANT Research Centre, University College Cork, Cork, Ireland; 3 National Perinatal Epidemiology Centre (NPEC), Department of Obstetrics and Gynaecology, University College Cork, Cork University Maternity Hospital, Cork, Ireland; 4 Health Behaviour Change Research Group, School of Psychology, NUI Galway, National University of Ireland, Galway, Ireland; 5 School of Public Health, University College Cork, Cork, Ireland

**Keywords:** Pregnancy, Weight management, Behaviour change, Facilitators, Barriers

## Abstract

**Background:**

Obesity and overweight are considered risk factors for a range of adverse outcomes, including stillbirth. This study aims to identify factors reported by women influencing weight management behaviours during pregnancy.

**Methods:**

A systematic search was conducted in five databases from inception to 2019 and updated in 2021. Qualitative studies involving pregnant or post-partum women, from high-income countries, examining women’s experiences of weight management during pregnancy were included. Meta-ethnography was used to facilitate the meta-synthesis of 17 studies.

**Results:**

Three themes were identified during the analysis: (1) Awareness and beliefs about weight gain and weight management, which included level of awareness and knowledge about dietary and exercise recommendations, risk perception and decision balance, perceived control over health and weight gain and personal insecurities. (2) Antenatal healthcare, women’s experiences of their interactions with healthcare professionals during the antenatal period and the quality of the education received had an effect on women’s behaviour. Further, our findings highlight the need for clear and direct information, and improved interactions with healthcare professionals, to better support women’s weight management behaviours. (3) Social and environmental influence, the social judgement and stigmatization associated with overweight and obesity also acted as a negative influence in womens’ engagement in weight management behaviours.

**Conclusion:**

Interventions developed to promote and maintain weight management behaviours during pregnancy should consider all levels of influence over women’s behaviours, including women’s level of awareness and beliefs, experiences in antenatal care, education provision and social influence.

**Supplementary Information:**

The online version contains supplementary material available at 10.1186/s12884-022-04929-z.

## Introduction

According to the World Health Organization (WHO), the prevalence of obesity worldwide has nearly tripled since 1975. Based on 2016 data, 39% of the adult population globally were overweight, and 13% had obesity [1]. A recent study, including 20 different European countries, concluded that 53.1% of their adult sample had overweight or obesity [2]. Increases in overweight and obesity and gestational weight gain trends are also observed amongst women of childbearing age (15 to 44 years old) [3, 4]. Overweight and obesity during pregnancy are associated with a wide range of complications including increased risk of gestational hypertension [5], preeclampsia [5], gestational diabetes mellitus [5], caesarean delivery [5], preterm birth [5], delivery of large-for-gestational infants [6], and stillbirth [7, 8]. Additionally, excess of gestational weight gain is associated with gestational diabetes, pregnancy-induced hypertension, caesarean delivery, postpartum weight retention, macrosomia and childhood obesity [9].

Weight management during pregnancy is therefore critically important and can involve individual behaviours related to diet and physical activity, as well as information and support from healthcare professionals. For the purpose of this study, we understand weight management as all of those behaviours that might influence women’s weight loss, weight maintenance and women’s gestational weight gain. Most information and advice that women receive about weight management during pregnancy is related to nutrition and levels of physical activity [10]. The NICE guidelines for example, recommend that pregnant women base meals on starchy and fibre-rich products, fruit and vegetables and eat a low-fat diet [11]. In terms of physical activity, the Royal College of Obstetricians and Gynaecologists (RCOG) and the Royal College of Physicians of Ireland (RCPI) recommend that women engage in a moderate amount of physical activity such as aerobic (e.g. swimming or running) or strength conditioning, starting with 10–15 min sessions for women who were not active before pregnancy, building up to 150 min per week [12, 13]. Comparison of prenatal physical activity guidelines from multiple high-income countries indicates that the majority recommend moderate physical activity, with recommendations to seek healthcare provider advice before starting or continuing an exercise program, and advice to not engage in sports that involve risk of falls, trauma or collision [14–16].

Weight management behaviours such as diet and physical activity are important because they are modifiable behaviours that women can engage in to improve outcomes for themselves and their infants. Despite the recognised benefits of a healthy diet and physical activity during pregnancy, previous studies have shown that a low proportion of pregnant women adhere to prenatal dietary guidelines and/or meet physical activity guidelines [17–19]. Caut et al. (2020) concluded from their systematic review that most preconceptual and pregnant women did not meet recommendations for vegetable, cereal grain or folate intake, and in half of the studies, women exceeded fat intake recommendations [19]. Regarding adherence to physical activity recommendations, a study conducted in the USA found that only 9.3% of pregnant women met the American College of Obstetricians and Gynaecologist (ACOG) guidelines for physical activity; 41% of women in this study reported engaging in physical activity < 1 day/week [17]. Such low levels of adherence to dietary and physical activity guidelines and recommendations highlights the need for greater understanding of women’s barriers and facilitators to engaging in these weight management behaviours.

Previous studies have explored experiences of pregnant women in relation to weight during pregnancy. Evidence of barriers and facilitators to gestational weight gain include physical barriers knowledge and beliefs, logistics and social barriers [20]. We believe that our study has a wider scope by exploring weight management which is understood as all of those behaviours that might have an influence on the women’s weight loss, weight maintenance or gestational weight. Identifying these facilitators and barriers can inform the development of interventions to better support women’s weight management during pregnancy and ultimately improve maternal and child health outcomes. Hence, the aim of this meta-synthesis is to identify and analyse qualitative research published to date in high income countries in order to establish what facilitators and barriers influence pregnant women’s weight management behaviours during pregnancy.

## Methods

The protocol for this meta-synthesis is registered on Prospero (no. CRD42019120069). Originally the objective of the registered meta-synthesis was to examine three different maternal behavioural risk factors for stillbirth (weight management behaviours, substance use, attendance at antenatal care). Given the complexity of these health behaviours and the large amount of literature exploring them, a synthesis including all three risk factors was deemed not to be feasible and/or likely to provide coherent findings across all behaviours. Consequently, this meta-synthesis differs from the protocol in that it focuses only on weight management behaviours rather than weight management, substance use and attendance at antenatal care. The findings in relation to the other two modifiable risk factors are published elsewhere [21, 22].

### Search strategy

We conducted a comprehensive search of the literature to obtain all qualitative studies that explored women’s facilitators and barriers to weight management during pregnancy. The databases searched were CINHAL, PsychINFO, Pubmed, SOCindex and Web of Science. Searches were conducted in March 2019 and updated in April 2021, with no restrictions on publication date.

Search terms used were facilitators, barriers, promoter, benefit, attitude, opportunity, determinant, promotion, intention, education, initiative, prevention, pregnancy, weight management, physical activity, nutrition, overweight (see example of search in Additional file 1).

### Study selection

The review of all titles and abstracts obtained from the search was completed independently by two members of the research team (TES, LL); three authors (TES, COC, KMS) conducted the full text screening.

Studies were included for further review if (1) they used a qualitative or a mixed methods design, as long as they included primary qualitative data; (2) they were written in English; (3) the participants were pregnant women or women up to 12 months post-partum as long as the data related to their pregnancy period (e.g. studies exploring post-partum weight management were excluded); (4) they were conducted in high-income countries; (5) they included extractable data about facilitators and barriers to manage weight during pregnancy; (6) they explored weight management behaviours that women were able to engage with personally (e.g. healthcare professionals’ weight management practices such as routine weighing were excluded).

Studies are restricted to high-income countries given the differences in care systems between high, middle, and low-income countries, and the different challenges associated with weight management in the different contexts. Furthermore, limiting the findings of this synthesis to high-income countries only will facilitate the development of prevention strategies applicable to high-income countries.

Studies that did not include any qualitative data or were not original research were excluded. Studies that included different types of participants (e.g. healthcare professionals, partners, or family members) were only included if the data obtained from the women was clearly differentiated from the data obtained from the other types of participants. Studies describing behaviours linked to weight management (such as diet and physical activity) but which were not explicitly linked to prenatal weight management in the published manuscript, were excluded.

### Data extraction

#### Study characteristics

A data extraction sheet was used to extract the characteristics of the studies by two authors (TES, COC). The following data were extracted from each study: country of publication, year of publication, aims, design, data collection method, sampling or recruitment strategy, consent process, number of participants, age of participants, pregnancy status, BMI and/or activity level of participants if applicable, timing of data collection, method of data analysis and study results.

#### Quality assessment

To assess study quality, the Critical Appraisal Skills Program for Qualitative studies checklist (CASP) [23] was used independently by three authors (TES, SM, COC). There are a number of different CASP checklist designed to asses different types of studies, including qualitative studies. The CASP Qualitative Studies Checklist – used in this study- is composed by ten different items exploring clarity of aims, adequacy of methodology and design, appropriateness of the recruitment strategy and data collection process, issues related to reflexivity and ethical considerations and clarity of the analytical process and statement of findings, as well as the value placed in the research. Since CASP does not provide a rating system, it was decided to rate as 0 for “No”, 1 for “Can’t Tell” and 2 for “Yes”, in order to facilitate grading of the studies.

A previous study conducted in the area of quality appraisals concluded that the quality of the reporting of a study correlates with its value towards the final synthesis, and that excluding studies with a low quality in their reporting will have very limited impact in the findings of the meta-synthesis [24]. Therefore, only studies with the highest quality (CASP ≥ 15) were part of our synthesis. Any discrepancies in ratings of quality appraisal using CASP tool were discussed between the members of the quality appraisal team to reach consensus based on the evidence provided in the papers.

Regarding the quality of our review findings, the GRADE-CERQual (Confidence in Evidence from Reviews of Qualitative research) approach was used. This approach facilitates assessing how much confidence can be placed in each individual review finding by evaluating four different components: methodological limitations, coherence, adequacy and relevance [25]. To conduct the GRACE-CERQual analysis, the iSoQ interactive summary of qualitative findings platform was used in its beta version [24].

#### Meta-ethnographic synthesis

To inform the synthesis of the included studies, a meta-ethnographic approach was used. Meta-ethnography is a qualitative methodology originally developed by Noblit and Hare with an interpretative approach that facilitates “putting together” the qualitative research available through a process of translating the studies into one another [25] and into the researcher’s own interpretation of the data. The use of meta-ethnography gives researchers the potential to produce new interpretations and models [26]. To facilitate the reporting of this meta-ethnography, we followed the eMERGe reporting guidance [27].

Noblit and Hare (1988) proposed a series of phases that overlap and repeat along with the conduction of the synthesis [25].

##### Phase 1 and 2 – Selecting meta-ethnography and deciding what is relevant

**Fig. 1:**
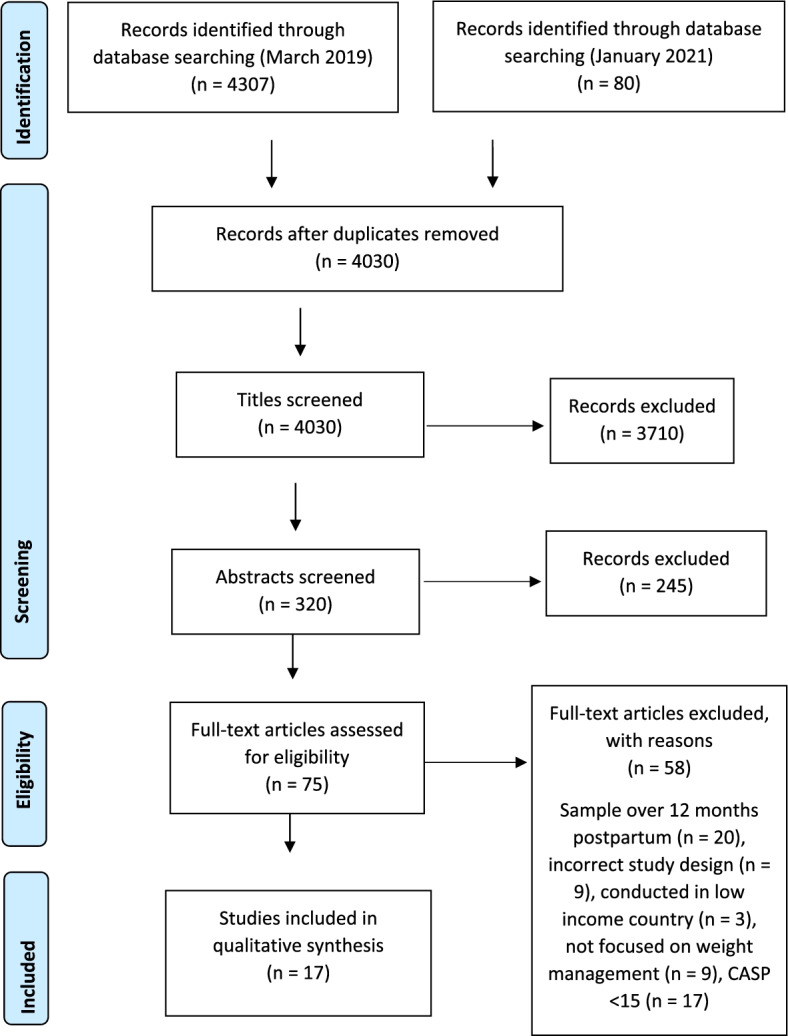
PRISMA Flow Diagram showing the process of inclusion of studies. From: Moher D, Liberati A, Tetzlaff J, Altman DG, The PRISMA Group (2009). Preferred Reporting Items for Systematic Reviews and Meta-Analyses: The PRISMA Statement. PLoS Med 6(7): e1000097. https://doi.org/10.1371/journal.pmed1000097

The first two phases of meta-ethnography involve identifying a research gap that might be fulfilled by meta-ethnography which was done above. Phase 2 was completed by performing a systematic search of the literature as reported above and importing the resulting studies into NVIVO12 for analysis (See Fig. 1).

##### Phase 3—Reading the studies

Phase 3 involved the repeated reading of the included studies by three reviewers (TES, CN, KMS) and the independent extraction of the characteristics and details of the study into the data extraction sheets (See Table 1). In this phase, the studies were read carefully, and notetaking facilitated the identification of the data that needed to be extracted or assessed for quality.

##### Phase 4—Determining how the studies are related

In order to identify common metaphors and concepts across the studies, line-by-line coding of the results and discussions section was conducted using NVIVO12 by one author (TES). The codes were refined as the coding progressed and new codes were created as necessary in each study. A second author (KMS) followed and checked the coding process to ensure its reliability and discuss nuances or disagreements.

Both first and second order constructs were extracted for analysis. In meta-ethnography, first order constructs are the participant quotes used by the original study’s authors, and second order constructs are the original authors interpretations reached after their own analysis of the data. These concepts are used to differentiate between the research participant’s experiences and the author’s interpretations of such experiences [28]. The influence of the author’s background over their interpretations done in the different papers was difficult to assess, as the reflexivity accounts of most studies were very poor or missing. The key concepts that we chose to explore and compare in the analysis were related to facilitators or barriers that influenced women’s weight management behaviours.

After completing the first coding phase, the content of each code was checked for consistency of interpretation and additional coding was performed when necessary. The resulting list of concepts was grouped into themes and categories through a thematic analysis process. We used tables to display the concepts and themes across all studies, classifying them into themes and categories, and then we used concept maps to establish and discuss the influences of each concept over the others.

##### Phase 5 – Translating studies into one another

In this phase, we continued to refine the themes and categories to ensure that the meaning of each individual study was reflected. The initial codes were examined, and themes were examined and combined thematically when describing similar findings.

The influence of each study over the different themes, categories and concepts identified in this synthesis is documented using references and quotes. Quotes were obtained from primary study participants and by primary author’s explanations and interpretations. Additionally, a table with a summary of the themes, categories and concepts identified is also provided (Additional file 2). In this article we are presenting first, second and third level interpretations, based on the women’s experiences.

##### Phase 6—Synthesizing translations

The result of the translated concepts, their relationships and the primary data were used to create a textual line of argument, which is presented in the results section. Two authors were involved in the synthesis (TES, KMS) and the additional authors provided feedback and insights when necessary (SM, CO, KOD, MB, LL). The authors are from different disciplinary backgrounds including psychology, sociology, medicine, public health, epidemiology and behavioural science, which promoted discussion of potential different interpretations.

##### Phase 7—Expressing the synthesis

The findings of this meta-ethnography are narratively presented in this article, additionally, a summarised version of the findings can be found in our CerQual assessment and summary tables (Additional file Table 2 and Additional file 3).

## Results

### Search outcomes

The first database search in March 2019, identified 4307 studies; the second search in January 2021 identified 80 additional records. Of these, 4030 remained after duplicate removal. After screening for titles and abstracts, 75 studies remained eligible for full text review. Following full text screening of the remaining 75 studies, 17 studies met the criteria for inclusion in the meta-synthesis (See Fig. 1).

### Study characteristics

Characteristics of included studies are shown in Table 1. Of the 17 studies included for analysis, four were conducted in the UK, six in the USA, three in Australia, three in Ireland, and one in Norway. The years of publication ranged from 2009 to 2020. The number of participants in the studies ranged from 11 to 58, with ages ranging from 17 to 46 years. Sixteen of the studies included pregnant women only, and one included both pregnant and postpartum women up to 12 months [29]. Fifteen studies used qualitative designs and two used mixed-methods designs. Twelve studies collected data using semi-structured interviews and five used focus groups. Regarding study quality, almost all studies performed poorly with regards to reflexivity, ethical considerations and reporting of their recruitment strategy. However, study quality was high due to clear aims, use of qualitative methodology, quality of report of findings and value of the research (Additional file Table 4). Results from the GRADE-CERQual analysis are presented in Additional file 3.

**Table 1:** Study characteristics

Authors	Country	Aims	Data collection method	Recruitment	Number of participants	Age	Pregnancy status	BMI required for participation	Timing of data collection	Method of data analysis	CASP score
Denison et al. (2015) [29]	UK	To explore the barriers and facilitators to physical activity and lifestyle interventions in pregnant women with Class III obesity (BMI >40kg/m2)	Semi-structured interviews	Purposive-sampling from a specialist-led clinic providing care to pregnant women with class III obesity.	13	25-34 years	Pregnant	Class III obesity (>40kg/m2)	17-37 weeks gestation	Framework approach	17
Ferrari et al. (2013) [30]	USA	Elicit from pregnant women their perceptions about provider advice regarding diet and physical activity in pregnancy.	Focus groups	Sample was part of a larger study. Recruited through newspaper ads, posted flyers and prenatal clinics.	58	18-35 years	Pregnant	N/A	27-30 weeks gestation	Thematic analysis	16
Faucher et al. (2020) [31]	UK	Identify beliefs and attitudes about GWG, exercise, and proposed intervention	Focus groups	Women were recruited with flyers and HCP approaches in their birth centres	17	17-35	Pregnant	Pre-pregnancy BMI of 30 or greater	<14 to 38 weeks gestation	Content Analysis	15
Flannery et al. (2020) [32]	Ireland	Explore overweight and obese women's experience and perception of dietary behaviours and weight management during pregnancy	Semi-structured interviews	Women with a BMI of 25 or higher were identified and recruited in a maternity hospital	30	20-40+	Pregnant	Between 20 and 40km/m2	Across all trimesters but most women between 27 and 40 weeks gestation.	Thematic analysis	19
Flannery et al. (2018) [33]	Ireland	Systematically identify the barriers and enablers to physical activity for women who are overweight and obese in pregnancy using the TDF and COM-B model.	Semi-structured interviews	Purposive-sampling of women with BMI ≥25kg/m2 in the public antenatal clinic of one maternity hospital.	30		Pregnant	BMI ≥25kg/m2	Across all trimesters	Framework approach with inductive thematic analysis	16
Garnweidner et al. (2013) [34]	Norway	To explore experiences with nutrition related information in routine antenatal care of an ethnically and socially diverse study population.	Semi-structured interviews	Recruited by midwives in eight mother and child health centres in Oslo.	17	average 28 years old	Pregnant and postpartum	Pre-pregnancy BMI ≥25kg/m2	Before the 30th week of pregnancy and two months afterwards	Interpretative phenomenological analysis	16
Groth et al. (2013) [35]	USA	Understand how urban, low income, pregnant African American women view physical activity and how they approach nutrition while pregnant.	Focus groups	Recruited from the Special Supplemental Nutrition Program for Women, Infants and Children (WIC) services and prenatal clinic waiting rooms	26	18-39 years	Pregnant	N/A	Over 60% in their first 20 weeks of pregnancy	Content analysis	15
Holton et al. (2017) [36]	Australia	Describe women's experiences and perspectives of care for weight management during pregnancy in Melbourne, Australia.	Semi-structured interviews	Recruited from Medical Centre using purposive recruitment strategies inc. Flyers and direct approaches from researchers	17	24-43 years (32.6 average)	Pregnant and postnatal	N/A	Late pregnancy (≥28 weeks gestation) and 4-6 weeks after giving birth.	Thematic analysis	15
Keely et al. (2017) [37]	UK	To explore the experiences, attitudes and health-related behaviours of pregnant women with a BMI >40kg/m2	Semi-structured interviews	Purposeful sampling in a specialist antenatal clinic for women with BMI >40kg/m2.	11	26-40 years	Pregnant and postnatal	Pregnant women with BMI ≥40kg/m2	2 interviews during pregnancy and 1 postnatally	Thematic content analysis	15
Kominiarek et al. (2015) [28]	USA	To examine and describe obese racial-ethnic minority women's knowledge, beliefs and attitudes about nutrition, exercise, and healthy lifestyles during pregnancy.	Focus-groups	Recruited at Women’s health clinic via recruitment flyers or recruited by study personnel.	16	21-39	Pregnant	Prepregnancy BMI ≥30kg/m2	Across all trimesters	Conventional qualitative data analysis (Thematic analysis)	17
Lee et al. (2018)* [38]	Australia	To assess and compare pregnancy nutrition recommendation knowledge and to explore how nutrition knowledge impacts on food choices in pregnant women and nutrition education practices of antenatal care providers.	Semi-structured interviews	Convenient sample from eligible pool of pregnant women attending the hospital for antenatal care. .	19	<30 to 40+	Pregnant	N/A	Across all trimesters, 45% women last trimester	Thematic analysis	15
Leiferman et al. (2011) [39]	USA	To elucidate unique barriers and facilitators to antenatal physical activity engagement among women of low socioeconomic status.	Individual and paired interviews	Passive print advertisement Via health care clinics and community organisations	25	18-46	Yes	N/A	Second and third trimester	Thematic analysis	15
Marquez et al. (2009) [40]	USA	To understand how Latina women perceived, understood, and valued exercise to inform a physical activity intervention designed.	Focus groups	Purposive sampling waiting room at the time of patients pre-natal appointments by bilingual/bicultural interviewers.	20	18 to 40	Yes	N/A	<28 weeks gestation	Content analysis	15
O'Brien et al. (2017) [41]	Ireland	To explore the various factors within the life course that overweight and obese pregnant women perceive to influence their food choice and physical activity behaviours.	semi-structured in depth interviews	Purposive sampling from patients attending the ODP	22	Mean 32.3	Yes	BMI ranging between 25kg/m2 and 39.9kg/m2	34th week of pregnancy	Inductive thematic analysis	16
Padmanabhan et al. (2015) [42]	UK	Examine pregnant women's weight-related attitudes and beliefs (including the weight-related behaviours of diet and physical activity during pregnancy).	Semi-structured face to face interviews	All participants were previously recruited to a prospective quantitative longitudinal study via invitation letters and participation information sheets.	19	19-38 years	Yes	N/A	Third trimester	systematic thematic content analysis	16
Reyes et al. (2013) [43]	USA	Understand the perceptions of low-income, overweight, and obese, African-American mothers about diet quality in pregnancy, specifically focused on what facilitators and barriers exist to eating healthy.	Semi-structured, individual interviews	Waiting room of a single university-affiliated outpatient prenatal care clinic, serving primarily Medicaid-insured patients.	21	≥18	Yes	Excluded in BMI <25	All trimesters in pregnancy	Principles of grounded theory	16
Sui et al. (2013)* [44]	Australia	Describe overweight and obese pregnant women's views about making healthy behavioural changes during pregnancy.	Face-to-face interviews	Purposeful sample of women participating in larger study.	26	not specified	Yes	Criteria of larger study.	28 weeks gestation	Framework analytical approach	16

^*^Mixed-methods studies

### Synthesis

Synthesis of the included papers led to three themes being generated: (1) Awareness, beliefs and emotions about weight management; (2) Antenatal healthcare; (3) Social and environmental influence (See Table 2).

**Table 2:** Summary of themes and categories

Themes	Categories
**Theme 1: Awareness, beliefs and emotions about weight gain and weight management**	1.1 Knowledge and awareness
	1.2 Risk perception and decision balance
	1.3 Perceived control over health and weight gain
	1.4 Personal insecurities and sensitive nature of the topic
**Theme 2: Antenatal healthcare**	2.1 Interactions with healthcare professionals
	2.2 Antenatal education and sources of information
**Theme 3: Social and environmental influence**	3.1 Influence of others, support and social norms
	3.2 Social judgement and stigmatization
	3.3 Environmental and sociodemographic factors

### Theme 1: Awareness and beliefs about weight gain and weight management

#### Category 1.1: Knowledge and awareness

Women included in ten reviewed studies discussed a general lack of knowledge about how to manage their weight, and many women were not aware of the appropriate range of gestational weight gain [28, 31, 32]. Women in nine studies demonstrated a general lack of awareness of the specific risks of living with overweight or obesity during pregnancy [28, 29, 31, 34, 36, 37, 42, 44].

“I don’t think there is a risk to me and my baby just yet. It’s just, I think, the number . . . I’ve gained 40 [pounds], so it’s not like a lot . . . I mean, even like you said, you gained 70 [pounds] but you were fine. So, the number I feel like is tricky.” [34] [First order construct]

Some women were not aware of the benefits of exercise [35, 41, 44] and healthy nutrition during pregnancy [38, 43, 44], with many women not aware what types or duration of physical activity is appropriate during pregnancy [29–31, 33, 34, 39, 40] or what types of food were recommended or considered healthy [38, 43, 44].

“I mean I don’t know can you do certain exercises so I would be worried that I could pull a muscle so I would be extra cautious I suppose at the gym cause I’m afraid and I wouldn’t really know” [33] [First order construct]

“I don’t know what contains iron in food.” [30] [First order construct]

Lack of knowledge and awareness was not universal and some women in two studies were aware of risks associated with being overweight pre-conception [38, 44], and knew recommendations for physical activity and nutritional during pregnancy in three studies [29, 39, 40].

“I’ve been warned by the midwife that I’ve been overweight and it’s so important that I should try to keep healthy.” [44] [First order construct]

Women’s knowledge and awareness about appropriate diet and physical activity during pregnancy [41, 43] was seen as linked to a range of benefits in thirteen studies [28–30, 33–35, 37–42, 44]. For instance, women reported that a healthy diet and physical activity could facilitate labour [29, 31, 39, 40, 42, 44], enhance feelings of control over their body [29, 39], improve baby’s well-being [39, 41, 43, 44], increase levels of energy [39], have general physical and mental health benefits [29, 39–42, 44], and facilitate social engagement with others [29, 39].

"Someone was comparing giving birth to a marathon…the more active you are and the more limber you are, then the easier it is to give birth." [29] [First order construct]

"They are both as important… diet because you are directly feeding the baby and you can control weight by eating the rights things. However, exercise…it's not only about weight control, it's about keeping your body moving and all sorts of other pregnancy things…" [29] [First order construct]

#### Category 1.2: Risk perception and decision balance

Our analysis suggests that women sometimes based their weight management behaviour decisions by weighing up advantages and disadvantages to the behaviours and assessing potential risks based on their knowledge. For instance, some women in two studies thought that risks associated with overweight or obesity during pregnancy are exaggerated [31, 44] and considered that the risks associated with other factors such as smoking or drinking alcohol are much higher [37, 42]. In addition, some women felt that eating in moderation justified their food choices [42] or that unhealthy choices can be [33, 37]. Whereas others perceived the pregnancy period as an excuse to have a treat [42] or overeat [28, 29, 31, 33, 35, 39, 40, 42, 44] as there was a perceived decrease in the pressure to lose weight and social perceptions such as the idea that women are “eating for two”. Some women also reported associating hunger with baby’s movements [42], as they believed that is the body’s way to express the baby’s nutritional needs [28, 31, 37, 42, 43]. Additionally, household work or active life was perceived by many women as enough activity to meet the physical activity recommendations in five of the studies [28, 29, 31, 35, 40].

“Oh, I think it feels like it gives you a free pass… I just think, ‘Well, I’m gonna.’…this is likely to be my last baby, I can lose the weight once I’m done” [37] [First order construct]

“I eat quite a lot of salad but I also eat quite a lot of chips and I know that chips are not healthy but I like them (laughs), in my mum’s house we eat a lot of veg and fruits, so I thought that was just enough really” [42] [First order construct]

“If she's moving, then it's like, ‘okay, well maybe she wants something’. When she moves, it's like, ‘maybe I'm hungrier than I feel. Maybe she needs something special.” [43] [First order construct]

“I know I don't really exercise but, like I said, getting the kids ready and walking to the car—I feel like that's sufficient enough for me to exercise." [28] [First order construct]

Perceived negative outcomes of behaviours were discussed by women in ten studies who expressed fears of harming the baby, having a premature birth or having a pregnancy loss while doing exercise [28, 29, 31, 33, 35, 39, 40, 42, 44]. For many women, feeling their body physically challenged was deemed as a risk [28, 42, 44] and so they decided to avoid exercise as their fears were stronger than the perceived benefits [42]. In two studies, women with previous fertility issues or history of pregnancy loss expressed that they were especially concerned about the safety of physical activity during pregnancy [33, 44].

‘I've seen pregnant people there and they're on the treadmill and I think ‘cool yourself'…you've got your baby bouncing up and down and then you've got your fat on top of the baby and it's just, you know, you could give them brain damage'." [29] [First order construct]

However, for women in three studies, while weight gain was acceptable during pregnancy, weight retention afterwards was not [28, 42, 44], which led to them engaging in weight management behaviours. Some women with more than one child discussed learning from their previous pregnancies and changing weight management habits in their current pregnancies to ensure they would lose any excess weight after birth [32, 39]. A feeling of satisfaction was also expressed by those women who saw the results of their efforts to manage their weight in their own body [42]. Some women in five studies felt a sense of responsibility for the health of their baby [30, 32, 41, 42, 44] which in some cases influenced their decision to prioritise healthy eating [31, 32, 41–43].

“Now because I only have a short time to go, I look at the scales and it’s a big achievement, and that’s brilliant, as what I have gained has been sufficient for the baby, but not to put on myself if you like, so I’m actually quite proud of myself" [42] [First order construct]

“Now I'm not eating a lot of greasy foods… (Before pregnancy) I wouldn't say I didn't care, but I got another life growing in me, so I don't want to jeopardize my life and the baby's life.” [43] [First order construct]

#### Category 1.3: Perceived control over health and weight gain

The findings show that women in three studies perceived limited control over their own health [30, 41], that pregnancy is risky by nature, and that complications occur randomly [37]. Some women also felt they did not have any control over their weight gain [28, 31, 42], which some women felt was a justification to “indulge” [28, 29, 32, 44]. Additionally, some women reported that their food choices were driven by cravings, appetite and taste [32, 35, 41–43].

“You can't control it, cuz that baby controlling it for you.” [28] [First order construct].

“According to our participants, biology and environment can conspire to make managing dietary intake feel beyond their control.” [31] [Second order construct]

Many of the women in fourteen studies experienced physical symptoms during pregnancy that were beyond their control, which acted as barriers to weight management [28–33, 35, 36, 39–44]. Barriers to physical activity included having a higher risk pregnancy [33, 40, 44], lack of energy and tiredness [29, 30, 33, 35, 39–44], reduced mobility [33, 39, 40], nausea, vomiting and other pregnancy related pains and discomforts [29, 33, 35, 36, 39, 40, 42, 44]. Barriers to a healthy diet included nausea, vomiting and aversion to certain foods [31, 32], lack of energy to cook40 or making food choices based on appetite and taste [32, 35, 41–43].

"I must admit a few weeks ago I tried [exercise] and I ended up in big trouble, I felt so sick, my head was swimming and my pelvis was killing and the baby wasn't happy and I thought no I've pushed, you know, when you've pushed it too far" [29] [First order construct]

“I think I gained weight due to severe morning sickness. The only thing I could eat was bread which helped to stop the nausea and heartburn. I ate bread even when I wasn’t hungry as it alleviated the alkaline taste on my tongue.” [36] [First order construct]

While women in four studies indicated they were aware of behavioural changes they were recommended to engage in, translating that awareness into action was experienced as a challenge [29, 35, 37, 44]. Women who lacked an established exercise routine [33, 35, 39, 40] or had poor pre-pregnancy dietary habits [28, 35, 37, 41, 43], experienced greater difficulties in changing their behaviours when becoming pregnant. This is also tied to motivation, and women in four different studies discussed lack of motivation as a barrier [29, 35, 39, 40] mostly for physical activity but also for healthy eating. Some women expressed that they disliked exercise [40, 44] or cooking [44], they felt too tired or ‘lazy’ [35, 39, 40] or that they needed an external motivation to remain active [35]. Conversely, women in six studies expressed that having established positive pre-pregnancy healthy habits acted as a facilitator to maintain these habits during pregnancy [33, 37, 40, 43, 44].

“ […] Like, I can’t do anything about it. I mean, I know all this… I mean I’ve studied this so much… like… I could be a dietitian probably! I just can’t implement it, for whatever reason, like… know what I mean?” [37] [First order construct]

“If I’ve already got an exercise routine, then stick with it… and just, if you are eating healthy, keep up with that.” [44] [First order construct]

Not all women experience lack of control, some women in two studies had a perceived high level of control over their weight gain in terms of their dietary intake and physical activity.

“I think it’s more because of my diet and everything and the way I am eating and I can actually see that you know it is working, not eating too much […] so I think that helps you know, that you can physically see that I am in control” [42] [First order construct]

### Theme 2: Antenatal healthcare

#### Category 2.1: Interactions with healthcare professionals

In this theme, we identified factors related to healthcare professionals’ attitudes that have an influence on women´s capacity and motivation to engage in weight management behaviours as expressed in five different studies [31–33, 37, 42]. Some women felt embarrassed and judged when dealing with healthcare professionals, especially with interactions regarding weight [32, 37]. Women reported insensitive and judgmental attitudes [37] and communication [32] from healthcare professionals, including in specialised clinics [31, 37]. Women felt they were assumed to have bad habits because of their overweight [37] or that they already had enough knowledge about weight management as they had children previously [33, 42].

"I had a very bad experience during my first pregnancy. I was 29 weeks and I went in to see my consultant and asked him if I could find out the sex of the baby but he just pinched my stomach … I felt very upset. I think they turn off when you are a little bit overweight. And they think oh she's after letting herself go.” [32] [First order construct]

"…what I found different was when they know that you have children already they kind of thinking that you know everything which is not true…you may forget […] but they seem to assume because you have had other children you know already what to do" [33] [First order construct]

Some women from four of the studies expressed defensive or avoidant attitudes that might potentially be a consequence of the negative feelings associated with overweight and the perception of weight management as a sensitive topic to address [30, 31, 34, 35]. This resulted in some women feeling challenged when receiving advice regarding their dietary behaviours. Some women in three studies considered BMI charts and ranges of weight gain as “a lie” [28], rejected standardised GWG goals [31], or expressed relief when the conversation about weight management with their providers was avoided [34].

"I was very frustrated my first pregnancy because my midwife was very keen on nutrition and, “Don’t gain too much weight,” and “We don’t want to have a really big baby.” And I wasn’t gaining any weight at all. And, so the fact that she was harping on it to me made me very angry […] It made me almost want to neglect the nutrition aspect, because I felt like she wasn’t listening to me personally." [31] [First order construct]

“I don't feel like I'm the typical obese person, you know. They say I'm obese, and I'm like, well, I don't know how you figure I'm obese. How do you classify obesity? I don't like that word because I don't feel—I know I'm big, but I'm not as big as most.” [28] [First order construct]

The feelings described by the women in this section, together with a perceived societal influence, were reflected in women’s perception that weight and weight management are sensitive topics to discuss in their antenatal care [28, 32, 34, 36, 37, 44]. As observed in six studies this acted as a barrier that minimised discussion opportunities and hindered knowledge exchange [28, 32, 34, 36, 37, 44]. However, some women from two studies felt that, even though it can be a difficult conversation, it is important to discuss weight and weight management with their healthcare professionals [28, 44].

"I feel very insecure about my weight … [the midwives] really tried not to mention it or make me feel uncomfortable." [35, 48] [First order construct]

"They reported that although it “hurt” when providers discussed their weight, they knew they were being truthful and ultimately it helped them." [37] [Second order construct]

However, finding supportive and non-judgemental healthcare professionals was also expressed by some women as important [34]. These women reported that sometimes they found it easier to speak to healthcare professionals about their weight management than speaking with the people in their close social context [34].

#### Category 2.2: Antenatal education and sources of information

Women included in eight studies discussed complaints about the availability of sources of information, or were not satisfied with the education and advice they received during their antenatal care [30, 32–34, 36, 38, 42, 44]. Women perceived that there was a lack of time to discuss weight management advice [30, 33, 36] or appropriate weight gain [28, 31–34, 36, 37, 42] during their antenatal care, and that clinical aspects were prioritised [32–34]. The information received about nutrition and diet while pregnant was perceived as scarce [34, 38], too generalised [30, 32, 34, 38, 42], and not tailored to individual needs [30, 34, 44], leaving women dissatisfied [30, 32–34, 36, 38, 42, 44]. Additionally, women reported that nutritional advice was focused on food safety only (i.e., preventing food-borne diseases), rather than weight management in four of the studies [32, 34, 36, 38]. Regarding physical activity during pregnancy, women also expressed dissatisfaction in seven of the studies [29, 30, 33, 36, 39, 42, 44]. The support received in relation to becoming more active was perceived as very limited [39, 44], and advice tended to be perceived as too conservative [30, 42], hesitant or unclear in recommendations [30, 33].

“They don’t tend to offer any advice good or bad in terms of weight management and activity and stuff like that. It’s more the blood pressure, checking the baby and stuff like that” [33] [First order construct]

“It was about the food I couldn’t eat. Like some types of raw fish and pasteurized milk and cheese, as I recall it?” [34] [First order construct]

“Nobody told me nothing [about physical activity]. They gave me some brochures [chuckling] and that's it.” [30] [First order construct]

Women in five studies felt that they were provided with useful nutrition and physical activity information during pregnancy, which acted as a facilitator to actively engaging in weight management [32, 34, 36, 38, 39]. Valuing and trusting providers opinions and advice was seen as beneficial in three studies [30, 34, 36].

The mentioned lack of discussion with healthcare professionals led some women to seek out other sources of information [32, 34, 38]. Some women found that having access to different sources of information was useful [29, 34, 38]. However, some women considered that the amount of information from the multitude of sources available was overwhelming [30, 34, 40, 42], especially in the cases where they would find conflicting or contradicting information [28, 30, 33, 38, 44].

“I saw a dietician at the pre-pregnancy clinic [due to diabetes]. She gave me useful information about food groups and healthy eating during pregnancy. I think other women would benefit from similar information.” [36] [First order construct]

“You know, it doesn’t say that much to me. It’s very, very much information you have to absorb during few consultations. I honestly have to admit that not all information is processed.” [34] [First order construct]

### Theme 3: Social and environmental influence

#### Category 3.1: Influence of others, support and social norms

In eight studies, women discussed how the dietary habits of their social circle influenced their beliefs and behaviours. In some instances, women reported that they had no decision power over shopping or cooking choices in their families [28, 31, 43], and felt the need to adapt their own nutrition habits to other people [28, 41]. Women in four studies also struggled when their family or partner encouraged them to overeat [28, 31, 32, 43] with some women describing their partners as ‘a feeder’ [32]. The misconception that women “need to eat for two” was shared amongst their wider circle. Some of the women in four studies reported that they relied on their family and friends for advice regarding weight management [28, 32, 34, 39]. This advice sometimes reinforced women’s own misconceptions, while some disagreed with advice they received [28, 34].

“The food that my mother buy [gets in the way of me reaching my GWG goal]. She don't buy healthy food, she, cuz my brother, and they all like fried chicken... She don't buy plain chicken.” [28] [First order construct]

“My family says eat as much as I want. And just keep eating, ‘Cause it’s good for the baby.” [43] [First order construct]

“I don't know what to follow. I don't know whether to obey what the nurse says about lying down or her [my husband's aunt].” [30] [First order construct]

For some women in seven studies, the lack of support and the lack of role models acted as a barrier to engage in physical activity and healthy eating [28, 29, 33, 39, 40, 42, 44]. Some of these women explained that their family members encouraged them to rest44, and even disapproved of their exercise [28]. However, availing of social support acted as a facilitator to engage in weight management behaviours for women in nine different studies [29, 32, 33, 35, 38–41, 44]. Having positive role models in their family who embedded positive physical activity and dietary habits in their childhood influenced their choices as adults [41].

“Lot of people have constantly said to me throughout my pregnancy, you need to rest, you need to rest, you need to rest. I don't really understand why I need to rest. If my body's not telling me that I need to rest you know, then why do I need to rest? … so it's been quite difficult.” [29] [First order construct]

“Well, just my husband and both my brothers, they all work out together, and me and my cousin work out, so everybody around me works out, so that kind of helped me.” [39] [First order construct]

Some cultural differences also acted as a barrier in three different studies [28, 34, 41]. In these studies, migrant women expressed how their cultural dietary habits or lay food beliefs did not match official health recommendations. Thus, advice was perceived as contradictory and challenging in some instances, especially when it involved additional family members with authority over the women [28, 34, 41].

"You know, that’s really weird, because in Norway you are told to eat eggs and fish, however in Pakistan you should stay away from it in the first three months of pregnancy." [34] [First order construct]

"You are in trouble when the elders say something and the midwife says something else. Especially your mother in law. She has much influence, especially during the first pregnancy. It is really difficult sometimes to decide what I should eat." [34] [First order construct]

#### Category 3.2: Social judgement and stigmatization

Many of the women that participated in ten studies had negative feelings about being overweight [28, 29, 33, 36–40, 42, 44]. Women expressed feelings of shame, guilt and regret for not being active [33, 37] or making food choices that they perceived as unhealthy [38]. For women in five studies, these feelings led them to have problems with their body-image and self-esteem [29, 36, 39, 40, 44].

“I beat myself up for it [weight gain]. I'm like, oh, I'm just so depressed, and I don't wanna eat no more.” [28] [First order construct]

“It’s going in like a swimming costume, it's definitely… Yeah, that's what puts me off 100%. It's nothing else… I'm like ‘oh my God,’ the thought of going swimming and people seeing me.’ [29] [First order construct]

Women discussed feeling social judgement and stigmatization in five studies [29, 36, 37, 41, 44], which acted as a barrier to engaging in weight management activities. Women felt they were treated differently if they were overweight [31, 36]; for instance, they felt questioned and judged as “greedy” [37, 42] or “lazy” [42], and reported that they had to hear to everyone’s opinion about their weight which was sometimes accompanied by derogatory language [42].

“When I was in the [hospital] waiting room, people looked at me differently … people think that you don’t look after yourself or take care of yourself when you are overweight.” [36] [First order construct]

“I think the thing as well…they think you’re just sitting here stuffing pints of Ben & Jerry's, like… that's not what my life is like…” [tearful]. [37] [First order construct]

#### Category 3.4: Environmental and sociodemographic factors

Regarding healthy eating, women in three studies reported that a lack of access to healthy food in their communities or workplaces acted as a barrier to engaging in dietary weight management [28, 43, 44]. Another barrier to healthy eating was the affordability and easy access to fast food in the women’s communities as reported in five studies [28, 31, 35, 41, 42], which led them to perceive healthy foods are more expensive and hard to access [41, 43]. Additionally, some women perceived that it was more convenient to buy ready meals than cooking at home [28, 35].

“I don't drive. So, when I get to the store, I have to go shopping for the entire month, because getting on the bus to go get groceries, it's too much.” [43] [First order construct]

"I think city life is probably not good for me... Takeaways and stuff, everything is delivered, you find stuff to do that's not even that active, like go to the cinema... it's just too easy to be bold" [41] [First order construct]

"It used to be expensive for Cornflakes and so cheap for porridge and it was always if you had less money you made you already made better choices... Now I don't know how they are doing chocolate and doughnuts so cheap... The people eating free--range eggs and wholegrain bread are middle class... They are educated people" [41] [First order construct]

Women also reported environmental barriers that influenced their capability to remain physically active during their pregnancies. Urban environments were considered unsuitable for outdoor exercise [41], especially in neighbourhoods that were perceived as unsafe [39, 40] or where the weather conditions were not suitable for outdoor sports [33, 39, 40, 42]. Women from five different studies expressed that they had very limited access to sports facilities [29, 39–42], and that the affordability of such facilities was a big barrier to exercise [29, 33, 39–42, 44]. Additionally, women reported that not having programmes tailored to pregnant women was an issue for them to engage in physical activity [29, 33, 40, 42].

“If you live in a bad neighborhood… it's not safe to exercise…You don't do that…” [40].

“I was looking for swimming baths…that I could afford [as unemployed]…I do like going to the gym. I have had to give up my gym membership because I'm not working and I can't afford it.” [29] [First order construct]

Many women from eight different studies expressed that they were not able to engage in weight management behaviours due to a lack of time [28, 29, 33, 35, 39–42]. For some women, their family and/or life acted as a barrier because it gave them limited time to cook and exercise [33, 38, 40, 42, 44].

“We tend to eat a lot of convenience food because I’m working full time and more things like fish fingers, chicken nuggets […] its always just whatever is in the freezer type of things” [42] [First order construct]

“I do find it, I would find it difficult to go out and do a proper exercise routine, because I just physically don’t have the time I don’t get in much until 6pm and I leave the house at 6am” [42] [First order construct]

Some socioeconomic factors were also associated with the barriers and facilitators that women reported regarding their weight management. In four studies reviewed, women living in low-income environments found additional barriers to manage their weight [28, 39–41], whereas women with higher educational level [34], higher socioeconomic status [41], multiparity and older age [41] reported more facilitators.

"I suppose just as the years progress, you know, you have to think for the future, because what you do right now will definitely benefit you for the future. And that's the way I look on it, you know. I think in your early twenties, you're out and what not and you know, your whole perspective on life completely changes" [41] [First order construct]

Some environment facilitators to exercise were discussed. Women reported that affordable facilities [33, 40], transportation and/or built environments suitable for physical activity, and having the right weather conditions acted as facilitators [39].

“I probably would be less active if they (trails, parks) weren’t there. I do walk up and down the neighbourhoods […] when I am really motivated I do the trail because the trail is long. But, yeah, I do think I would be less active if I didn’t have that (trail).” [39] [First order construct]

## Discussion

In this synthesis, we identified factors that act as barriers and facilitators to women’s willingness and ability to engage in weight management behaviours during pregnancy, which in this review were mostly related to physical activity and following dietary advice. The main themes identified were: (1) Awareness and beliefs about weight management, (2) Antenatal healthcare, and (3) Social and environmental influence. The main barriers identified were a lack of knowledge about the risks of overweight and obesity during pregnancy and lack of awareness about the recommendations regarding physical activity and diet. Women who had pre-established unhealthy habits experienced additional challenges to adopting recommendations during pregnancy, whereas women who already had healthy habits found it easier to maintain these habits. Antenatal healthcare experiences also influenced women’s behaviour. Women who had negative interactions with healthcare professionals felt shamed and stigmatised; this made weight management discussions with their healthcare professionals problematic and acted as a barrier to engaging in weight management behaviours. Further, women reported a perceived lack of prioritisation of weight management during their antenatal care from their healthcare professionals, which led them to feel like the discussions were rushed and the advice very scarce or limited. On the other hand, having adequate awareness of the recommendations regarding physical activity and diet during pregnancy, as well as how to engage in these behaviours safely acted as facilitators. Women who were aware of the potential risks of being overweight during pregnancy and that had a high level of perceived control over their health were also more keen to engage in weight management behaviours. Additionally, encountering supportive healthcare professional and having access to sources of information and discussion was also positive for women. Additionally, women’s social contexts also played an important role in their engagement in weight management behaviours.

Our findings suggest that women’s level of knowledge and awareness regarding weight management recommendations influences their engagement and this is in line with previous findings that found women who were informed about physical activity held more favourable attitudes towards it [45]. This lack of knowledge might be contributing to misconceptions about the risks of engaging in weight management behaviours during pregnancy, which together with the collective perceived fragility of pregnancies, led women to have increased concerns and fears regarding the safety of their pregnancies.

Lack of knowledge and awareness about the recommendations regarding healthy nutrition and physical activity during pregnancy might also contribute to women’s perceived low self-efficacy. This is in concordance with previous research conducted in the Netherlands examining socio-cognitive determinants of physical activity, which concluded that possessing knowledge and skills to engage in healthy behaviours improves confidence to overcome other types of barriers, and increases the perceived benefits of changing behaviours [46]. Additionally, our findings suggest that women perceive dietary advice to be predominantly focused on food-borne illness prevention rather than weight management. This might be related to healthcare professionals need to prioritise safety when giving nutritional advice to pregnant women. Although it has been shown in previous research that information provision alone is insufficient to produce behaviour change [47], providing women with clear, concise and practical information regarding physical activity and healthy eating during pregnancy might not only contribute to temper such misconceptions and highlight the benefits of these behaviours, but it might also increase the women’s perceived control over their own health, as well as their levels of self-efficacy and motivation.

Findings of our review indicate that social stigma associated with overweight and obesity in general, and especially during pregnancy, led some women and healthcare professionals to feel that weight management is a sensitive topic to discuss. Consequently, some women responded with avoidant attitudes and some others adopted a defensive response. The NICE guidelines for weight management during pregnancy suggest that healthcare professionals discuss dietary and physical activity habits with women at the first opportunity to address concerns and myths, and advise women about the benefits of physical activity and healthy nutrition during pregnancy [11]. However, a previous study exploring healthcare professionals approach to weight management identified that their perceived stigma and consequent “cautious approach”[48] were interfering in the care provided by healthcare professionals [48] in concordance with our review findings. These findings are also supported by previous examinations indicating that professionals from a range of specialities reported that weight is a difficult topic to discuss with potential to damage their relationship with their patients, and sometimes represents an inappropriate use of their time [49, 50]. Such attitudes might influence the quality of information that women are receiving, as the conversation with HCP regarding weight management in our findings has been shown to be quite limited. This lack of discussion with healthcare professionals about weight management during pregnancy also likely contributes to women’s lack of knowledge about aspects of weight management. This in turn led some women to resort to other sources of information, which women reported to include conflicting information. Providing trustworthy sources of information and encouraging discussion about weight management during pregnancy with healthcare professionals is important to overcome this barrier.

Previous research demonstrates that unhealthy social eating environments are a risk factor for obesity [51]. Based on our findings, issues such as the influence of the family context, the lack of support and role models, or the influence of social norms and stigmatisation of overweight during pregnancy will have an influence on women’s engagement in weight management behaviours. Additionally, physical environments play a role in women’s engagement in weight management behaviours during pregnancy, as shown in our findings. The quick and easy access to fast food and lack of access to affordable exercise facilities reported by women were probably contributing to women’s low engagement in weight management behaviours, as also shown in previous research [52]. Previous studies have shown how altering elements of people’s environments helped modulate their behaviour [53]. Decisions at policy level regarding free access to exercise facilities for pregnant women and/or promoting barriers to accessing fast food such as price increases or location regulation would be very beneficial. As such, interventions at individual and population levels may enhance women’s engagement in health promoting and weight management behaviours during pregnancy.

Interventions need to be designed taking into consideration all the different levels of influence over women’s weight management behaviours. The factors identified in this synthesis speak to inter-related influences and levels, which can be understood using the socioecological model. This model states that there is no one single factor influencing people’s health, and it sees health levels as the interaction among many factors at five different levels [54]. The five different levels are 1) individual, including knowledge, attitude and skills; 2) interpersonal, including family, friends and social networks; 3) organizational, including social institutions; 4) community, including relationship between organizations and 5) public policy, including national, state, local and legal regulations[54]. Hence, this framework highlights the importance of including all these levels in intervention design in order to address all the social determinants of health involved in a certain issue.

Our themes are similar to findings exposed in the literature [55, 56]. However, we believe that our analysis has been able to identify further nuances into the facilitators and barriers. Additionally, our study differs from the ones conducted before in that we are not only interested in women’s experiences of gestational weight gain, but in pregnant women’s experiences of engaging with weight management behaviours to either loose excessive weight or manage their gestational weight gain, and also, it is not focused on a specific type of population (e.g.: ethnic group, etc.).

There are limitations to this study. Firstly, the number of databases used for this study was limited; however we chose the selected databases based on appropriateness with the topic and similar studies in the area. Secondly, to ensure our findings were derived from robust, high-quality studies, we excluded some studies based on quality standards. While this might have contributed to losing some relevant input, we are confident that the findings presented are from high quality primary evidence. Thirdly, the findings resulting from this synthesis are based on women’s experiences and opinions, and do not include partner’s, healthcare professionals’ or policy makers’ perspectives on the issue; further research should be conducted to explore facilitators and barriers from other stakeholder’s points of view on this issue. Additionally, our findings are a result of a secondary analysis of primary data, which was collected and analysed by the studies’ primary authors. This means that our own background might potentially have an influence on the review findings. To address this potential limitation, we have tried to remain transparent about the influence of each study over the different findings and conducted a GRADE CERQual assessment of confidence in our review findings.

## Conclusion

Our review findings show that women’s weight management behaviours are influenced at multiple levels during pregnancy. Pre-established habits have shown to be important predictors of whether women adopt recommendations during pregnancy, hence, interventions to reduce obesogenic environments and generally promote healthy habits in relation to physical activity and diet at a population level have the potential to also benefit women during pregnancy. Additionally, women would benefit from clear and direct sources of information and improved discussion with healthcare professionals. Development of interventions to foster and maintain weight management behaviours during pregnancy should consider women’s awareness and beliefs, habits and motivation levels, social influence and their experiences in antenatal care. Such interventions should also include family members or other people in women’s social circles to facilitate engagement and adherence. Provision of additional training regarding the complexities of weight management during pregnancy for healthcare professionals would also help them to further develop key practical and emotional skills to better support the pregnant women under their care to engage in appropriate weight management behaviours. Such approaches are needed to minimise adverse pregnancy outcomes of high gestational weight gain for women and infants.

## Supplementary Information

**Additional file 1. **

**Additional file 2.**

**Additional file 3.**

**Additional file 4.**

## Data Availability

The data supporting the conclusions of this article is available in these published articles: Denison et al. [29], Ferrari et al. [30], Faucher et al. [31], Flannery et al. [32], Flannery et al. [33], Garnweidner et al. [34], Groth et al. [35], Holton et al. [36], Keely et al. [37], Kominiarek et al. [28], Lee et al. [38], Leiferman et al. [39], Marquez et al. [40], O'Brien et al. [41], Padmanabhan et al. [42], Reyes et al. [43], Sui et al. [44].
