# Supplementary material for: Facilitators and barriers influencing weight management behaviours during pregnancy: a meta-synthesis of qualitative research
Source: BMC Pregnancy Childbirth. 2022 Sep 5;22:682. doi: 10.1186/s12884-022-04929-z (PMC9443069; doi:10.1186/s12884-022-04929-z)
Supplement: Supplementary file 1 — Additional file 1. [file 12884_2022_4929_MOESM1_ESM.docx]

| **Database: Web of Science**  **Date:** | |
| --- | --- |
|  | **Search Terms** |
| **Concept 1 Facilitators and Barriers** | |
|  | TO Facilitator* |
|  | TO Barrier* |
|  | TO Promoter* |
|  | TO Benefit* |
|  | TO Attitude* |
|  | TO Opportunit* |
|  | TO Determinant* |
|  | TO Promotion |
|  | TO Intention |
|  | TO Education |
|  | TO Initiative |
|  | TO Prevention |
|  | TO Pregnancy |
|  | **S1 OR S2 OR S3 OR S4 OR S5 OR S6 OR S7 OR S8 OR S9 OR S10 OR S11 OR S12 AND S13** |
| **Concept 5 Weight Management** | |
|  | TO Weight management |
|  | TO Physical activity |
|  | TO Nutrition |
|  | TO Overweight |
|  | **S33 OR S34 OR S35** |
|  | **S14 AND S19** |
| **Notes**  Limiters: English Language | |

Supplementary file 1. Example of search strategy
