# Supplementary material for: Facilitators and barriers influencing weight management behaviours during pregnancy: a meta-synthesis of qualitative research
Source: BMC Pregnancy Childbirth. 2022 Sep 5;22:682. doi: 10.1186/s12884-022-04929-z (PMC9443069; doi:10.1186/s12884-022-04929-z)
Supplement: Supplementary file 2 — Additional file 2. [file 12884_2022_4929_MOESM2_ESM.docx]

| **Themes** | Facilitators | Barriers |
| --- | --- | --- |
| Theme 1: Awareness and beliefs about weight gain and weight management | **Knowledge and awareness**   - Awareness of physical activity recommendations.^1–3^ - Awareness of risks of overweight and underweight during pregnancy.^4,5^ - Awareness of health benefits of healthy eating and exercise^1,2,11–13,3–10^ – easier labour^1–3,5,13,14^, feeling of control over pregnant body^1,2^, prevent excessive GWG^4^,   improve baby’s well-being^2,5,12,15^, increased energy^2^, enjoyable activities^2,3^, physical and mental health benefits^1–3,5,12,13^, socialisation^1,2^.  **Risk perception and decision balance**   - Having different attitude in second and subsequent pregnancies regarding exercise and nutrition^2,16^. - Positively accepting weight gain during pregnancy^13^ - Prioritising food quality^12,15^ - Concerns about gaining too much weight^5,11,13^ - Sense of responsibility towards baby^5,6,12,13,16^ - Becoming a role children for their children^2,10,12^ - Prioritising healthy eating because of pregnancy^12–16^   **Perceived control over health and weight gain**   - Established exercise habit before and during pregnancy^7^ - Established healthy diet habits before and during pregnancy^3,10,15^ - Motivation   - Intrinsic motivation^2^   - Perceived high levels of self-efficacy and confidence^12^ - Considering oneself able despite pregnancy^11^. | **Knowledge and awareness**   - Lack of awareness of benefits of exercise during pregnancy^5,9,12^ - Lack of awareness on how to safely exercise^1–3,6–8,14^ - Lack of nutrition related knowledge – benefits and what’s healthy^4,5,15^ - Lack of awareness of appropriate range of GWG^11,14,16^ - Lack of awareness of specific risks of excessive weight gain^1,5,8,10,11,13,14,17^   **Risk perception and decision balance**   - Not feeling susceptible to risks – considering risks exaggerated^5,14^ - Perception of higher risk associated with smoking or alcohol drinking^10,13^ - Perception that pregnancy is risky by nature and complications happen randomly^10^. - Perceiving pregnancy as free pass period to eat anything or overeat^7,10,11,13–17^ - Idea that unhealthy choices can be compensated^13^ – intention to delay improving health habits until postpartum period^7,10^ - Considering household works as exercise enough^1,3,9,11,14^ - Considering physical activity as unsafe^1–3,5,7,9,11,13,14^ - Fear of harming the baby while doing exercise^1–3,5,7,9,11,13,14^ (mention body physically challenged^3^) - Fear of harming the baby by eating too little^14,15^.   **Perceived control over health and weight gain**   - Perceived lack of control over GWG^11,13,14^ - Acceptance that weight gain is inevitable, excuse to indulge^1,5,11,16^ - Perception that own control of health is very limited^6,12^ - Justifying unhealthy choices by eating in moderation^13^ - Understanding cravings as nutritional needs of baby^10,11,13–15^ - Pregnancy related barriers to exercise   - Being restricted by HCP / higher risk pregnancies^3,5,7^   - Lack of energy, tiredness^1–3,5–7,9,12,13,15^   - Reduced mobility^2,3,7^   - Sickness and other pregnancy related pains and discomforts^1–3,5,7,9,13,17^ - Pregnancy related barriers to healthy diet   - Nausea, vomiting and aversion to certain foods^14,16^   - Making food choices based on cravings, appetite and taste^9,12,13,15,16^   - Lack of energy to cook^15^ - Lack of exercise routine and poor dietary habits - Habit of eating out^9^ - Snacking^15^ - Using food as comfort in times of distress^10–12^ - Lack of established exercise routine^2,3,7,9^ - Lack of motivation^1–3,9^   - Dislike of exercise^3,5^   - Laziness^2,3,9^   - Need for external motivation^9^   **Personal insecurities and sensitive nature of the topic**   - Feelings of shame and guilt - Shame and regret for not being active^7,10^ - Guilt for choosing unhealthy foods^4^ - Shame because of overweight – problems with body-image, self-steem^1–3,5,17^. - Perceived need to justify dietary behaviours^13^   Weight and diet perceived as sensitive topic for both women and healthcare professionals^5,8,10,11,16,17^. |
| Theme 2: Antenatal healthcare | **Interaction with Healthcare Professionals**  Supportive healthcare professionals^8^  **Antenatal education and sources of information**  Advice from healthcare professionals   - Receiving useful information about nutrition and exercise^2,4,8,16,17^ - Valuing and trusting providers opinions, information and advice^6,8,17^.   Culturally sensitive information^8,11^  Access to different sources of information^1,4,8^ | **Interaction with healthcare professionals**  Insensitive and judgemental attitudes^16^   - Feeling embarrassed and ashamed by insensitive comments^16^ - Feeling stigmatised in dietary specialist clinics^10,14^ - Judgemental attitudes from HCP^10^ - Insensitive and inappropriate communication^16^   Avoidant or defensive attitudes   - Feeling challenged when receiving advice about nutrition^6,9,14^. - Personal disagreement with HCP advice^6^. - Rejecting standards GWG goals^14^. - Actively avoiding conversation about weight^8^.   **Antenatal education and sources of information**   - Receiving conflicting information from different sources^4–7,11^ - Too much information-too many sources^3,6,8,13^ - Perceived lack of prioritising of exercise and nutrition advice^6,7,17^ – prioritising of clinical aspects^7,8,16^ - Lack of pre-conceptual education^5^ - Using anecdotal evidence to reduce anxiety^10,14^   Education received about physical activity   - Lack of satisfaction with information received^1,2,5–7,13,17^ - Perceived lack of support to become more active^2,5^ - Perceiving physical activity advice as being too conservative^6,13^ - Having to prompt discussion themselves^6^ - Hesitant and unclear recommendations^6,7^   Education received about diet and nutrition   - Insufficient, scarce advice^4,8^ - Lack of discussion with HCP about dietary advice^4,6–8,13^ - Advice too generalised – not especific^4,6,8,13,16^ - Advice not individualised^5,6,8^ - Nutritional advice focused on food safety only^4,8,16,17^ - Eating advice confusing and changing – difficulties trying to meet all dietary advice^6^ - Having to prompt discussion themselves^8^ |
| Theme 3: Social and environmental influence | **Availability of support**   - Supportive and encouraging partner, friends and family^1–5,7,9,12,16^ - Having positive role models^12^   **Environmental and sociodemographic facilitators**   - Having good cooking skills^4^ - Affordable facilities^3,7^ - Availability of transportation or built environment^2^ - Right weather conditions^2,3^ - High educational level - Higher socioeconomic status - Multiparty and older age^12^ | **Influence of others, social support and norms**   - Adapting own dietary habits to others^11,12^ - Lack of control over shopping or cooking choices^11,14,15^ - Cultural differences in dietary habits^8,11,12^ - Family encouraging overeating^11,14–16^ - Unhealthy habits in supportive circle^11^ - Relying on family for advice^2,8,11,16^ - Lay advice contradicting professional advice^8,11^ - Social pressure to increase self-soothing eating^11,17^ - Lack of support and role model^1–3,5,7,11,13^ - Social perception of exercise being unsafe^11^ - Family and partner encouraging inactivity^3^ - Relying on family for advice (both) ^2,8,11,16^ - Lay advice contradicting professional advice (both) ^8,11^   **Social judgement and stigmatization**^1,5,10,12,17^   - Perceived differentiated treatment compared to other pregnant women^14,17^ - Feeling questions and judged^12–14^   Being exposed to derogatory language^13^  **Environmental and sociodemographic barriers**   - Affordability and easy access to fast food^9,11–14^ - Healthy food perceived as more expensive and harder to access - Lack of cooking skills^12^ - lack of nearby healthy shops^5,11,15^ - Lack of exercise programs tailored to pregnant women^1,3,7,13^ - Limited access to sports facilities – affordability issues^1–3,5,7,12,13^ - Neighbourhood safety^2,3^ - Urban environments unsuitable for outdoor exercise^12^ - Weather conditions^2,3,7,13^ - Low income environments^11^ |

1. Denison, F. C., Weir, Z., Carver, H., Norman, J. E. & Reynolds, R. M. Physical activity in pregnant women with Class III obesity: A qualitative exploration of attitudes and behaviours. *Midwifery* **31**, 1163–1167 (2015).

2. Leiferman, J., Swibas, T., Koiness, K., Marshall, J. A. & Dunn, A. L. My Baby, My Move: Examination of Perceived Barriers and Motivating Factors Related to Antenatal Physical Activity. *J. Midwifery Women’s Heal.* **56**, 33–40 (2011).

3. Marquez, D. X. *et al.* Perspectives of Latina and Non-Latina White Women on Barriers and Facilitators to Exercise in Pregnancy. *Women Health* **49**, 505–521 (2009).

4. Lee, A., Newton, M., Radcliffe, J. & Belski, R. Pregnancy nutrition knowledge and experiences of pregnant women and antenatal care clinicians: A mixed methods approach. *Women and Birth* **31**, 269–277 (2018).

5. Sui, Z., Turnbull, D. A. & Dodd, J. M. Overweight and obese women’s perceptions about making healthy change during pregnancy: A mixed method study. *Matern. Child Health J.* **17**, 1879–1887 (2013).

6. Ferrari, R., Siega-Riz, A., Everson, K., Moos, M. & Carrier, K. A qualitative study of women’s perceptions of provider advice about diet and physical activity. **91**, 372–377 (2014).

7. Flannery, C. *et al.* Enablers and barriers to physical activity in overweight and obese pregnant women: An analysis informed by the theoretical domains framework and COM-B model. *BMC Pregnancy Childbirth* **18**, 1–13 (2018).

8. Garnweidner, L. M., Sverre Pettersen, K. & Mosdøl, A. Experiences with nutrition-related information during antenatal care of pregnant women of different ethnic backgrounds residing in the area of Oslo, Norway. *Midwifery* **29**, e130–e137 (2013).

9. Groth, S. W., Morrison-Beedy, D. & Morrison‐Beedy, D. Low‐income, Pregnant, African American Women’s Views on Physical Activity and Diet. *J. Midwifery Women’s Heal.* **58**, 195–202 (2013).

10. Keely, A., Cunningham-Burley, S., Elliott, L., Sandall, J. & Whittaker, A. “If she wants to eat…and eat and eat…fine! It’s gonna feed the baby”: Pregnant women and partners’ perceptions and experiences of pregnancy with a BMI >40 kg/m2. *Midwifery* **49**, 87–94 (2017).

11. Kominiarek, M. A., Gay, F. & Peacock, N. Obesity in Pregnancy: A Qualitative Approach to Inform an Intervention for Patients and Providers. *Matern. Child Health J.* **19**, 1698–1712 (2015).

12. O’Brien, O. A. *et al.* Influences on the food choices and physical activity behaviours of overweight and obese pregnant women: A qualitative study. *Midwifery* **47**, 28–35 (2017).

13. Padmanabhan, U., Summerbell, C. D. & Heslehurst, N. A qualitative study exploring pregnant women’s weight-related attitudes and beliefs in UK: The BLOOM study. *BMC Pregnancy Childbirth* **15**, 1–14 (2015).

14. Faucher, M. A. & Mirabito, A. M. Pregnant Women with Obesity Have Unique Perceptions About Gestational Weight Gain, Exercise, and Support for Behavior Change. *J. Midwifery Womens. Health* **65**, 529–537 (2020).

15. Reyes, N. R., Klotz, A. A. & Herring, S. J. A Qualitative Study of Motivators and Barriers to Healthy Eating in Pregnancy for Low-Income, Overweight, African-American Mothers. *J. Acad. Nutr. Diet.* **113**, 1175–1181 (2013).

16. Flannery, C. *et al.* Dietary behaviours and weight management: A thematic analysis of pregnant women’s perceptions. *Matern. Child Nutr.* **16**, 1–10 (2020).

17. Holton, S., East, C. & Fisher, J. Weight management during pregnancy: a qualitative study of women’s and care providers’ experiences and perspectives. *BMC Pregnancy Childbirth* **17**, 351 (2017).
