# Supplementary material for: Facilitators and barriers influencing weight management behaviours during pregnancy: a meta-synthesis of qualitative research
Source: BMC Pregnancy Childbirth. 2022 Sep 5;22:682. doi: 10.1186/s12884-022-04929-z (PMC9443069; doi:10.1186/s12884-022-04929-z)
Supplement: Supplementary file 3 — Additional file 3. [file 12884_2022_4929_MOESM3_ESM.docx]

## **Summary of Qualitative Findings Table**

| # | **Summarised review finding** | **GRADE-CERQual Assessment of confidence** | **Explanation of GRADE-CERQual Assessment** | **References** |
| --- | --- | --- | --- | --- |
| 1 | Some women lacked knowledge regarding how to manage their weight during pregnancy. This lack of knowledge included lack of awareness of risks, and lack of awareness of recommendations regarding physical activity and dietary advice and the associated benefits. | High confidence | Minor concerns regarding methodological limitations, No/Very minor concerns regarding coherence, No/Very minor concerns regarding adequacy, and No/Very minor concerns regarding relevance | Faucher & Mirabito undefined; Groth et al. undefined; Denison et al. undefined; Flannery et al. undefined; Garnweidner et al. undefined; Reyes et al. undefined; Lee et al. undefined; Sui et al. undefined; O'Brien et al. undefined; Padmanabhan et al. undefined; Keely et al. undefined; Holton et al. undefined; Kominiarek et al. undefined; |
| 2 | Understanding the value of food quality and benefits of physical activity during pregnancy, as well as being aware of the recommendations, acted as facilitator for some women. | High confidence | Minor concerns regarding methodological limitations, Minor concerns regarding coherence, No/Very minor concerns regarding adequacy, and No/Very minor concerns regarding relevance | Faucher & Mirabito undefined; Groth et al. undefined; Denison et al. undefined; Flannery et al. undefined; Garnweidner et al. undefined; Reyes et al. undefined; Lee et al. undefined; Leiferman et al. undefined; Sui et al. undefined; Flannery et al. undefined; O'Brien et al. undefined; Ferrari et al. undefined; Marquez et al. undefined; Padmanabhan et al. undefined; Keely et al. undefined; Kominiarek et al. undefined; |
| 3 | Women engaged in a process of weighing up perceived benefits and potential risks to make choices about their weight management behaviours. Some women perceived the risks of overweight and obesity as exaggerated and felt that unhealthy choices could be compensated. | Moderate confidence | Minor concerns regarding methodological limitations, Minor concerns regarding coherence, No/Very minor concerns regarding adequacy, and No/Very minor concerns regarding relevance | Faucher & Mirabito undefined; Groth et al. undefined; Denison et al. undefined; Flannery et al. undefined; Reyes et al. undefined; Leiferman et al. undefined; Sui et al. undefined; Flannery et al. undefined; O'Brien et al. undefined; Marquez et al. undefined; Padmanabhan et al. undefined; Keely et al. undefined; Holton et al. undefined; Kominiarek et al. undefined; |
| 4 | Some women expressed concerns of harming their babies when doing exercise, especially in the cases of women with fertility issues or history of pregnancy loss. These fears led women to believe that the potential risks were stronger than the benefits of physical activity, so they decided to avoid it. | High confidence | Minor concerns regarding methodological limitations, No/Very minor concerns regarding coherence, No/Very minor concerns regarding adequacy, and Minor concerns regarding relevance | Groth et al. undefined; Denison et al. undefined; Reyes et al. undefined; Leiferman et al. undefined; Sui et al. undefined; Flannery et al. undefined; Marquez et al. undefined; Padmanabhan et al. undefined; Kominiarek et al. undefined; |
| 5 | Some women expressed feelings of shame and guilt due to their weight. These negative feelings had a pejorative effect over their body-image and self-steem which prevented them from wanting to engage in physical activities. Additionally, such feelings also acted as a barrier for communication and information transmission between women and healthcare professionals as weight management was perceived as a sensitive topic. | High confidence | Minor concerns regarding methodological limitations, Minor concerns regarding coherence, No/Very minor concerns regarding adequacy, and No/Very minor concerns regarding relevance | Faucher & Mirabito undefined; Denison et al. undefined; Flannery et al. undefined; Garnweidner et al. undefined; Lee et al. undefined; Leiferman et al. undefined; Sui et al. undefined; Flannery et al. undefined; O'Brien et al. undefined; Marquez et al. undefined; Padmanabhan et al. undefined; Keely et al. undefined; Holton et al. undefined; Kominiarek et al. undefined; |
| 6 | Women’s pre-pregnancy habits played an important role in their weight management behaviours during pregnancy. Women who lacked an exercise routine or had poor dietary habits encountered additional challenges, whereas women who had healthy pre-established habits found it easier to maintain them. | High confidence | Minor concerns regarding methodological limitations, No/Very minor concerns regarding coherence, Minor concerns regarding adequacy, and No/Very minor concerns regarding relevance | Groth et al. undefined; Reyes et al. undefined; Leiferman et al. undefined; Flannery et al. undefined; O'Brien et al. undefined; Marquez et al. undefined; Keely et al. undefined; Kominiarek et al. undefined; |
| 7 | Lack of motivation to healthy eating or physical activity were reported as common barriers which could be overcome with the influence of external motivations. | Moderate confidence | Moderate concerns regarding methodological limitations, Minor concerns regarding coherence, Moderate concerns regarding adequacy, and No/Very minor concerns regarding relevance | Groth et al. undefined; Denison et al. undefined; Leiferman et al. undefined; Sui et al. undefined; Marquez et al. undefined; |
| 8 | Pregnancy symtpoms like tiredness, reduced mobility, or nausea can act as barriers to physical activity and interfere in women’s eating behaviours because of potential aversion to foods, cravings, or levels of appetite. | High confidence | Minor concerns regarding methodological limitations, No/Very minor concerns regarding coherence, No/Very minor concerns regarding adequacy, and No/Very minor concerns regarding relevance | Faucher & Mirabito undefined; Groth et al. undefined; Denison et al. undefined; Flannery et al. undefined; Reyes et al. undefined; Leiferman et al. undefined; Sui et al. undefined; Flannery et al. undefined; O'Brien et al. undefined; Ferrari et al. undefined; Marquez et al. undefined; Padmanabhan et al. undefined; Holton et al. undefined; |
| 9 | For some women, being pregnant acted as a motivation to make changes in their habits towards healthier diet and activity behaviours. These women reported that felt a sense of responsibility towards the wellbeing of their babies, they wanted to be healthy for their children and act as role model for their own health behaviours. | High confidence | Minor concerns regarding methodological limitations, No/Very minor concerns regarding coherence, No/Very minor concerns regarding adequacy, and No/Very minor concerns regarding relevance | Faucher & Mirabito undefined; Flannery et al. undefined; Reyes et al. undefined; Leiferman et al. undefined; Sui et al. undefined; O'Brien et al. undefined; Ferrari et al. undefined; Padmanabhan et al. undefined; Keely et al. undefined; |
| 10 | The social context of the woman has an influence in their weight management behaviours. Some women reported having very limited control over shopping or cooking options, or were exposed to family members who encouraged overeating. Additionally, family members acted as a source of information or advice that sometimes contradicted HCP recommendations. | High confidence | Minor concerns regarding methodological limitations, No/Very minor concerns regarding coherence, No/Very minor concerns regarding adequacy, and No/Very minor concerns regarding relevance | Faucher & Mirabito undefined; Flannery et al. undefined; Garnweidner et al. undefined; Reyes et al. undefined; Leiferman et al. undefined; O'Brien et al. undefined; Holton et al. undefined; Kominiarek et al. undefined; |
| 11 | The lack of support from family and friends to engage in weight management behaviours acted as a barrier for many women. In some occasions, these women lacked positive role models and were encouraged to rest. | High confidence | Minor concerns regarding methodological limitations, No/Very minor concerns regarding coherence, No/Very minor concerns regarding adequacy, and No/Very minor concerns regarding relevance | Denison et al. undefined; Flannery et al. undefined; Garnweidner et al. undefined; Leiferman et al. undefined; Sui et al. undefined; Flannery et al. undefined; Marquez et al. undefined; Padmanabhan et al. undefined; Kominiarek et al. undefined; |
| 12 | Having the support of the social context to engage in weight managements behaviours acted as facilitators, especially when women had positive role models throughout all their lifes. | High confidence | Minor concerns regarding methodological limitations, No/Very minor concerns regarding coherence, No/Very minor concerns regarding adequacy, and No/Very minor concerns regarding relevance | Groth et al. undefined; Denison et al. undefined; Flannery et al. undefined; Lee et al. undefined; Sui et al. undefined; Flannery et al. undefined; O'Brien et al. undefined; Marquez et al. undefined; |
| 13 | Some women expressed feeling judged and stigmatised for being overweight and pregnant. These negative feelings contribute to the perception that weight management is a sensitive topic to discuss, preventing information exchange and advice provision with HCP. | Moderate confidence | Minor concerns regarding methodological limitations, No/Very minor concerns regarding coherence, Minor concerns regarding adequacy, and No/Very minor concerns regarding relevance | Faucher & Mirabito undefined; Denison et al. undefined; Sui et al. undefined; O'Brien et al. undefined; Padmanabhan et al. undefined; Keely et al. undefined; Holton et al. undefined; |
| 14 | Some women encountered insensitive or judgemental HCP during their antenatal care that made them feel embarrassed and stigmatised for being overweight. These led some women to adopt avoidant attitudes, where they avoided to speak about weight management, or defensive attitudes, where they received advice regarding weight management as a challenge to their individuality. | Low confidence | Minor concerns regarding methodological limitations, Minor concerns regarding coherence, Moderate concerns regarding adequacy, and Moderate concerns regarding relevance | Faucher & Mirabito undefined; Flannery et al. undefined; Keely et al. undefined; |
| 15 | Women were not satisfied the level of education received regarding weight management during their antenatal care. Some women felt overwhelmed by the amount of information and sources available to them, especially regarding dietary behaviours, which led them to find contradictive information in some cases. Information was too hesitant or conservative, and lacked specific advice. | High confidence | Minor concerns regarding methodological limitations, No/Very minor concerns regarding coherence, No/Very minor concerns regarding adequacy, and No/Very minor concerns regarding relevance | Denison et al. undefined; Flannery et al. undefined; Garnweidner et al. undefined; Lee et al. undefined; Leiferman et al. undefined; Sui et al. undefined; Ferrari et al. undefined; Padmanabhan et al. undefined; Holton et al. undefined; |
| 16 | Women who received information and advice regarding physical activity and diet during pregnancy were more able to engage in weight management behaviours. These women valued their HCP opinions and engaged actively in information seeking behaviours. | Moderate confidence | Minor concerns regarding methodological limitations, Moderate concerns regarding coherence, Minor concerns regarding adequacy, and No/Very minor concerns regarding relevance | Flannery et al. undefined; Garnweidner et al. undefined; Lee et al. undefined; Leiferman et al. undefined; Ferrari et al. undefined; Holton et al. undefined; |
| 17 | Environmental factors such as accessibility to healthy shops or sports facilities and affordability of fast food, and lack of time or other commitments also had a negative influence on women’s physical activity and dietary habits. | High confidence | Minor concerns regarding methodological limitations, No/Very minor concerns regarding coherence, No/Very minor concerns regarding adequacy, and No/Very minor concerns regarding relevance | Faucher & Mirabito undefined; Groth et al. undefined; Denison et al. undefined; Reyes et al. undefined; Lee et al. undefined; Leiferman et al. undefined; Sui et al. undefined; Flannery et al. undefined; O'Brien et al. undefined; Marquez et al. undefined; Padmanabhan et al. undefined; Kominiarek et al. undefined; |
| 18 | Women living in low-income environments found additional barriers to manage their weigh, whereas women with higher educational level, higher socioeconomic status, multiparity and older age reported more facilitators. | High confidence | Minor concerns regarding methodological limitations, No/Very minor concerns regarding coherence, Minor concerns regarding adequacy, and No/Very minor concerns regarding relevance | Garnweidner et al. undefined; Leiferman et al. undefined; O'Brien et al. undefined; Marquez et al. undefined; Kominiarek et al. undefined; |

# **GRADE-CERQual Assessment Worksheet**

# **Evidence Profile**

| **#** | **Summarized Review Finding** | **Methodological limitations** | **Coherence** | **Adequacy** | **Relevance** | **GRADE-CERQual assessment of confidence** | **References** |
| --- | --- | --- | --- | --- | --- | --- | --- |
| 1 | Some women lacked knowledge regarding how to manage their weight during pregnancy. This lack of knowledge included lack of awareness of risks, and lack of awareness of recommendations regarding physical activity and dietary advice and the associated benefits. | **Minor concerns**  Minor concerns regarding methodological limitations because poor reporting of reflexibity statement and ethical concerns. | **No/Very minor concerns**  The data supporting this finding is quite descriptive, good fit between the finding and the data | **No/Very minor concerns**  This finding is supported by a high number of studies with rich data | **No/Very minor concerns**  A variety of high-income countries are represented in the studies contributing to this findings. | **High confidence**  Minor concerns regarding methodological limitations, No/Very minor concerns regarding coherence, No/Very minor concerns regarding adequacy, and No/Very minor concerns regarding relevance | Denison et al. undefined;  Faucher & Mirabito undefined;  Flannery et al. undefined;  Garnweidner et al. undefined;  Groth et al. undefined;  Holton et al. undefined;  Keely et al. undefined;  Kominiarek et al. undefined;  Lee et al. undefined;  O'Brien et al. undefined;  Padmanabhan et al. undefined;  Reyes et al. undefined;  Sui et al. undefined; |
| 2 | Understanding the value of food quality and benefits of physical activity during pregnancy, as well as being aware of the recommendations, acted as facilitator for some women. | **Minor concerns**  **Minor concerns regarding methodological limitations because poor reporting of reflexibity statement and ethical concerns.** | **Minor concerns**  **Minor concerns regarding coherence because some data shows that awareness is not always translated into behaviour change.** | **No/Very minor concerns**  **All studies except one contribute to this finding** | **No/Very minor concerns**  **All studies except one contribute to this finding** | **High confidence**  **Minor concerns regarding methodological limitations, Minor concerns regarding coherence, No/Very minor concerns regarding adequacy, and No/Very minor concerns regarding relevance** | Denison et al. undefined;  Faucher & Mirabito undefined;  Ferrari et al. undefined;  Flannery et al. undefined;  Flannery et al. undefined;  Garnweidner et al. undefined;  Groth et al. undefined;  Keely et al. undefined;  Kominiarek et al. undefined;  Lee et al. undefined;  Leiferman et al. undefined;  Marquez et al. undefined;  O'Brien et al. undefined;  Padmanabhan et al. undefined;  Reyes et al. undefined;  Sui et al. undefined; |
| 3 | Women engaged in a process of weighing up perceived benefits and potential risks to make choices about their weight management behaviours. Some women perceived the risks of overweight and obesity as exaggerated and felt that unhealthy choices could be compensated. | **Minor concerns**  Minor concerns regarding methodological limitations because poor description of recruitment strategy overall and poor description of reflexivity statements and ethical issues. | **Minor concerns**  Minor concerns regarding coherence because this finding is highly interpretative but there is enough data to support it. | **No/Very minor concerns**  The number of studies and richness of data is enough to support finding | **No/Very minor concerns**  Wide range of contexts represented, direct relevance | **Moderate confidence**  Minor concerns regarding methodological limitations, Minor concerns regarding coherence, No/Very minor concerns regarding adequacy, and No/Very minor concerns regarding relevance | Denison et al. undefined;  Faucher & Mirabito undefined;  Flannery et al. undefined;  Flannery et al. undefined;  Groth et al. undefined;  Holton et al. undefined;  Keely et al. undefined;  Kominiarek et al. undefined;  Leiferman et al. undefined;  Marquez et al. undefined;  O'Brien et al. undefined;  Padmanabhan et al. undefined;  Reyes et al. undefined;  Sui et al. undefined; |
| 4 | Some women expressed concerns of harming their babies when doing exercise, especially in the cases of women with fertility issues or history of pregnancy loss. These fears led women to believe that the potential risks were stronger than the benefits of physical activity, so they decided to avoid it. | **Minor concerns**  Minor concerns regarding methodological limitations because poor description of recruitment strategy overall and poor description of reflexivity statements and ethical issues. | **No/Very minor concerns**  No or very minor concerns. No data contradicting the finding. | **No/Very minor concerns**  The number of studies and richness of data is enough to support finding | **Minor concerns**  Minor concerns regarding relevance because 6 out of the 9 studies are specific to USA context. | **High confidence**  Minor concerns regarding methodological limitations, No/Very minor concerns regarding coherence, No/Very minor concerns regarding adequacy, and Minor concerns regarding relevance | Denison et al. undefined;  Flannery et al. undefined;  Groth et al. undefined;  Kominiarek et al. undefined;  Leiferman et al. undefined;  Marquez et al. undefined;  Padmanabhan et al. undefined;  Reyes et al. undefined;  Sui et al. undefined; |
| 5 | Some women expressed feelings of shame and guilt due to their weight. These negative feelings had a pejorative effect over their body-image and self-steem which prevented them from wanting to engage in physical activities. Additionally, such feelings also acted as a barrier for communication and information transmission between women and healthcare professionals as weight management was perceived as a sensitive topic. | **Minor concerns**  Minor concerns regarding methodological limitations because because of poor description of recruitment strategy overall and poor description of reflexivity statements and ethical issues. | **Minor concerns**  Minor concerns regarding coherence because this finding is highly interpretative. Although there is enough data to support the finding, slightly different interpretations might be possible. | **No/Very minor concerns**  The number of studies and richness of data is enough to support finding | **No/Very minor concerns**  Wide range of contexts represented, direct relevance to review question | **High confidence**  Minor concerns regarding methodological limitations, Minor concerns regarding coherence, No/Very minor concerns regarding adequacy, and No/Very minor concerns regarding relevance | Denison et al. undefined;  Faucher & Mirabito undefined;  Flannery et al. undefined;  Flannery et al. undefined;  Garnweidner et al. undefined;  Holton et al. undefined;  Keely et al. undefined;  Kominiarek et al. undefined;  Lee et al. undefined;  Leiferman et al. undefined;  Marquez et al. undefined;  O'Brien et al. undefined;  Padmanabhan et al. undefined;  Sui et al. undefined; |
| 6 | Women’s pre-pregnancy habits played an important role in their weight management behaviours during pregnancy. Women who lacked an exercise routine or had poor dietary habits encountered additional challenges, whereas women who had healthy pre-established habits found it easier to maintain them. | **Minor concerns**  Minor concerns regarding methodological limitations because because of poor description of recruitment strategy overall and poor description of reflexivity statements and ethical issues. | **No/Very minor concerns**  No or very minor concerns. No data contradicting the finding. | **Minor concerns**  Minor concerns regarding adequacy because only 8 of 17 studies are contributing to this finding, however data is rich enough | **No/Very minor concerns**  Wide range of contexts represented, direct relevance | **High confidence**  Minor concerns regarding methodological limitations, No/Very minor concerns regarding coherence, Minor concerns regarding adequacy, and No/Very minor concerns regarding relevance | Flannery et al. undefined;  Groth et al. undefined;  Keely et al. undefined;  Kominiarek et al. undefined;  Leiferman et al. undefined;  Marquez et al. undefined;  O'Brien et al. undefined;  Reyes et al. undefined; |
| 7 | Lack of motivation to healthy eating or physical activity were reported as common barriers which could be overcome with the influence of external motivations. | **Moderate concerns**  Moderate concerns regarding methodological limitations because of poor description of reflexivity statements and ethical issues | **Minor concerns**  Minor concerns regarding coherence because no data contradicting the finding. | **Moderate concerns**  Moderate concerns regarding adequacy because of the small number of studies supporting this finding | **No/Very minor concerns**  Wide range of contexts and sociodemographoc range represented, direct relevance | **Moderate confidence**  Moderate concerns regarding methodological limitations, Minor concerns regarding coherence, Moderate concerns regarding adequacy, and No/Very minor concerns regarding relevance | Denison et al. undefined;  Groth et al. undefined;  Leiferman et al. undefined;  Marquez et al. undefined;  Sui et al. undefined; |
| 8 | Pregnancy symtpoms like tiredness, reduced mobility, or nausea can act as barriers to physical activity and interfere in women’s eating behaviours because of potential aversion to foods, cravings, or levels of appetite. | **Minor concerns**  Minor concerns regarding methodological limitations because of poor description of recruitment strategy overall and poor description of reflexivity statements and ethical issues. | **No/Very minor concerns**  No data contradicting the finding. | **No/Very minor concerns**  The number of studies and richness of data is enough to support finding | **No/Very minor concerns**  Wide range of contexts represented, direct relevance to research question | **High confidence**  Minor concerns regarding methodological limitations, No/Very minor concerns regarding coherence, No/Very minor concerns regarding adequacy, and No/Very minor concerns regarding relevance | Denison et al. undefined;  Faucher & Mirabito undefined;  Ferrari et al. undefined;  Flannery et al. undefined;  Flannery et al. undefined;  Groth et al. undefined;  Holton et al. undefined;  Leiferman et al. undefined;  Marquez et al. undefined;  O'Brien et al. undefined;  Padmanabhan et al. undefined;  Reyes et al. undefined;  Sui et al. undefined; |
| 9 | For some women, being pregnant acted as a motivation to make changes in their habits towards healthier diet and activity behaviours. These women reported that felt a sense of responsibility towards the wellbeing of their babies, they wanted to be healthy for their children and act as role model for their own health behaviours. | **Minor concerns**  Minor concerns regarding methodological limitations because of poor description of recruitment strategy overall and poor description of reflexivity statements and ethical issues. | **No/Very minor concerns**  No data contradicting the finding. Good fit between data and finding. | **No/Very minor concerns**  The number of studies and richness of data is enough to support finding | **No/Very minor concerns**  Wide range of contexts represented, direct relevance to research question | **High confidence**  Minor concerns regarding methodological limitations, No/Very minor concerns regarding coherence, No/Very minor concerns regarding adequacy, and No/Very minor concerns regarding relevance | Faucher & Mirabito undefined;  Ferrari et al. undefined;  Flannery et al. undefined;  Keely et al. undefined;  Leiferman et al. undefined;  O'Brien et al. undefined;  Padmanabhan et al. undefined;  Reyes et al. undefined;  Sui et al. undefined; |
| 10 | Having the support of the social context to engage in weight managements behaviours acted as facilitators, especially when women had positive role models throughout all their lifes. | **Minor concerns**  Minor concerns regarding methodological limitations because of poor description of recruitment strategy overall and poor description of reflexivity statements and ethical issues. | **No/Very minor concerns**  No data contradicting the finding. Good fit between data and finding. | **No/Very minor concerns**  The number of studies and richness of data is enough to support finding | **No/Very minor concerns**  Wide range of contexts represented, direct relevance to research question | **High confidence**  Minor concerns regarding methodological limitations, No/Very minor concerns regarding coherence, No/Very minor concerns regarding adequacy, and No/Very minor concerns regarding relevance | Denison et al. undefined;  Flannery et al. undefined;  Flannery et al. undefined;  Groth et al. undefined;  Lee et al. undefined;  Marquez et al. undefined;  O'Brien et al. undefined;  Sui et al. undefined; |
| 11 | The lack of support from family and friends to engage in weight management behaviours acted as a barrier for many women. In some occasions, these women lacked positive role models and were encouraged to rest. | **Minor concerns**  Minor concerns regarding methodological limitations because of poor description of recruitment strategy overall and poor description of reflexivity statements and ethical issues. | **No/Very minor concerns**  No data contradicting the finding. Good fit between data and finding. | **No/Very minor concerns**  The number of studies and richness of data is enough to support finding | **No/Very minor concerns**  The number of studies and richness of data is enough to support finding | **High confidence**  Minor concerns regarding methodological limitations, No/Very minor concerns regarding coherence, No/Very minor concerns regarding adequacy, and No/Very minor concerns regarding relevance | Denison et al. undefined;  Flannery et al. undefined;  Flannery et al. undefined;  Garnweidner et al. undefined;  Kominiarek et al. undefined;  Leiferman et al. undefined;  Marquez et al. undefined;  Padmanabhan et al. undefined;  Sui et al. undefined; |
| 12 | Having the support of the social context to engage in weight managements behaviours acted as facilitators, especially when women had positive role models throughout all their lifes. | **Minor concerns**  Minor concerns regarding methodological limitations because of poor description of recruitment strategy overall and poor description of reflexivity statements and ethical issues. | **No/Very minor concerns**  No data contradicting the finding. Good fit between data and finding. | **No/Very minor concerns**  The number of studies and richness of data is enough to support finding | **No/Very minor concerns**  Wide range of contexts represented, direct relevance to research question | **High confidence**  Minor concerns regarding methodological limitations, No/Very minor concerns regarding coherence, No/Very minor concerns regarding adequacy, and No/Very minor concerns regarding relevance | Denison et al. undefined;  Flannery et al. undefined;  Flannery et al. undefined;  Groth et al. undefined;  Lee et al. undefined;  Marquez et al. undefined;  O'Brien et al. undefined;  Sui et al. undefined; |
| 13 | Some women expressed feeling judged and stigmatised for being overweight and pregnant. These negative feelings contribute to the perception that weight management is a sensitive topic to discuss, preventing information exchange and advice provision with HCP. | **Minor concerns**  Minor concerns regarding methodological limitations because of poor description of recruitment strategy overall and poor description of reflexivity statements and ethical issues. | **No/Very minor concerns**  No data contradicting the finding. Good fit between data and finding. | **Minor concerns**  Minor concerns regarding adequacy because of the limited number of studies supporting this finding | **No/Very minor concerns**  Wide range of contexts represented, direct relevance to research question | **Moderate confidence**  Minor concerns regarding methodological limitations, No/Very minor concerns regarding coherence, Minor concerns regarding adequacy, and No/Very minor concerns regarding relevance | Denison et al. undefined;  Faucher & Mirabito undefined;  Holton et al. undefined;  Keely et al. undefined;  O'Brien et al. undefined;  Padmanabhan et al. undefined;  Sui et al. undefined; |
| 14 | Some women encountered insensitive or judgemental HCP during their antenatal care that made them feel embarrassed and stigmatised for being overweight. These led some women to adopt avoidant attitudes, where they avoided to speak about weight management, or defensive attitudes, where they received advice regarding weight management as a challenge to their individuality. | **Minor concerns**  Minor concerns regarding methodological limitations because of poor description of recruitment strategy overall and poor description of reflexivity statements and ethical issues. | **Minor concerns**  Minor concerns regarding coherence because this finding is highly interpretative. Although there is enough data to support the finding, slightly different interpretations might be possible. | **Moderate concerns**  Moderate concerns regarding adequacy because very limited number of studies supporting this finding | **Moderate concerns**  Moderate concerns regarding relevance because context limited to UK and Ireland | **Low confidence**  Minor concerns regarding methodological limitations, Minor concerns regarding coherence, Moderate concerns regarding adequacy, and Moderate concerns regarding relevance | Faucher & Mirabito undefined;  Flannery et al. undefined;  Keely et al. undefined; |
| 15 | Women were not satisfied the level of education received regarding weight management during their antenatal care. Some women felt overwhelmed by the amount of information and sources available to them, especially regarding dietary behaviours, which led them to find contradictive information in some cases. Information was too hesitant or conservative, and lacked specific advice. | **Minor concerns**  Minor concerns regarding methodological limitations because of poor description of recruitment strategy overall and poor description of reflexivity statements and ethical issues. | **No/Very minor concerns**  No data contradicting the finding. Good fit between data and finding. | **No/Very minor concerns**  The number of studies and richness of data is enough to support finding | **No/Very minor concerns**  Wide range of contexts represented, direct relevance to research question | **High confidence**  Minor concerns regarding methodological limitations, No/Very minor concerns regarding coherence, No/Very minor concerns regarding adequacy, and No/Very minor concerns regarding relevance | Denison et al. undefined;  Ferrari et al. undefined;  Flannery et al. undefined;  Garnweidner et al. undefined;  Holton et al. undefined;  Lee et al. undefined;  Leiferman et al. undefined;  Padmanabhan et al. undefined;  Sui et al. undefined; |
| 16 | Women who received information and advice regarding physical activity and diet during pregnancy were more able to engage in weight management behaviours. These women valued their HCP opinions and engaged actively in information seeking behaviours. | **Minor concerns**  Minor concerns regarding methodological limitations because of poor description of recruitment strategy overall and poor description of reflexivity statements and ethical issues. | **Moderate concerns**  Moderate concerns regarding coherence because of limited data contradicting trustworthiness of healthcare professionals | **Minor concerns**  Minor concerns regarding adequacy because of limited number of studies supporting this finding. | **No/Very minor concerns**  Wide range of contexts represented, direct relevance to research question | **Moderate confidence**  Minor concerns regarding methodological limitations, Moderate concerns regarding coherence, Minor concerns regarding adequacy, and No/Very minor concerns regarding relevance | Ferrari et al. undefined;  Flannery et al. undefined;  Garnweidner et al. undefined;  Holton et al. undefined;  Lee et al. undefined;  Leiferman et al. undefined; |
| 17 | Environmental factors such as accessibility to healthy shops or sports facilities and affordability of fast food, and lack of time or other commitments also had a negative influence on women’s physical activity and dietary habits. | **Minor concerns**  Minor concerns regarding methodological limitations because of poor description of recruitment strategy overall and poor description of reflexivity statements and ethical issues. | **No/Very minor concerns**  No data contradicting the finding. Good fit between data and finding. | **No/Very minor concerns**  The number of studies and richness of data is enough to support finding | **No/Very minor concerns**  Wide range of contexts represented, direct relevance to research question | **High confidence**  Minor concerns regarding methodological limitations, No/Very minor concerns regarding coherence, No/Very minor concerns regarding adequacy, and No/Very minor concerns regarding relevance | Denison et al. undefined;  Faucher & Mirabito undefined;  Flannery et al. undefined;  Groth et al. undefined;  Kominiarek et al. undefined;  Lee et al. undefined;  Leiferman et al. undefined;  Marquez et al. undefined;  O'Brien et al. undefined;  Padmanabhan et al. undefined;  Reyes et al. undefined;  Sui et al. undefined; |
| 18 | Women living in low-income environments found additional barriers to manage their weigh, whereas women with higher educational level, higher socioeconomic status, multiparity and older age reported more facilitators. | **Minor concerns**  Minor concerns regarding methodological limitations because of poor description of recruitment strategy overall and poor description of reflexivity statements and ethical issues. | **No/Very minor concerns**  No data contradicting the finding. Good fit between data and finding. | **Minor concerns**  Minor concerns regarding adequacy because of the limited number of studies supporting the finding | **No/Very minor concerns**  Wide range of contexts represented, direct relevance to research question | **High confidence**  Minor concerns regarding methodological limitations, No/Very minor concerns regarding coherence, Minor concerns regarding adequacy, and No/Very minor concerns regarding relevance | Garnweidner et al. undefined;  Kominiarek et al. undefined;  Leiferman et al. undefined;  Marquez et al. undefined;  O'Brien et al. undefined; |
