# Supplementary material for: Facilitators and barriers influencing weight management behaviours during pregnancy: a meta-synthesis of qualitative research
Source: BMC Pregnancy Childbirth. 2022 Sep 5;22:682. doi: 10.1186/s12884-022-04929-z (PMC9443069; doi:10.1186/s12884-022-04929-z)
Supplement: Supplementary file 4 — Additional file 4. [file 12884_2022_4929_MOESM4_ESM.docx]

| Reference | Clear aims | Qualitative methodology appropiate | Research design appropiate to meet aims | Recruitment strategy appropiate | Data collected in a way that addressed the research issue | Relationship between researcher and participants considered | Ethical issues | Data analysis rigorous | clear statement of findings | How valuable is the research | Total Score |
| --- | --- | --- | --- | --- | --- | --- | --- | --- | --- | --- | --- |
| Denison et al. | 2 | 2 | 2 | 2 | 2 | 0 | 1 | 2 | 2 | 2 | 17 |
| Ferrari et al. | 2 | 2 | 1 | 2 | 2 | 0 | 1 | 2 | 2 | 2 | 16 |
| Faucher et al. | 2 | 1 | 2 | 1 | 2 | 1 | 1 | 1 | 2 | 2 | 15 |
| Flannery et al. | 2 | 2 | 2 | 1 | 2 | 2 | 2 | 2 | 2 | 2 | 19 |
| Flannery et al. | 2 | 2 | 1 | 2 | 2 | 0 | 1 | 2 | 2 | 2 | 16 |
| Garnweidner et al. | 2 | 2 | 2 | 1 | 2 | 0 | 1 | 2 | 2 | 2 | 16 |
| Groth et al. | 2 | 2 | 2 | 1 | 2 | 0 | 1 | 1 | 2 | 2 | 15 |
| Holton et al. | 2 | 2 | 2 | 1 | 2 | 0 | 0 | 2 | 2 | 2 | 15 |
| Keely et al. | 2 | 2 | 2 | 2 | 1 | 1 | 1 | 1 | 2 | 1 | 15 |
| Kominiarek et al. | 2 | 2 | 2 | 1 | 2 | 1 | 1 | 2 | 2 | 2 | 17 |
| Lee et al. | 2 | 2 | 2 | 1 | 1 | 0 | 1 | 2 | 2 | 2 | 15 |
| Leiferman et al. | 2 | 2 | 2 | 1 | 1 | 0 | 1 | 2 | 2 | 2 | 15 |
| Marquez et al. | 2 | 2 | 2 | 1 | 2 | 0 | 1 | 2 | 2 | 1 | 15 |
| O'Brien et al. | 2 | 2 | 1 | 2 | 2 | 1 | 1 | 1 | 2 | 2 | 16 |
| Padmanabhan et al. | 2 | 2 | 2 | 1 | 2 | 0 | 1 | 2 | 2 | 2 | 16 |
| Reyes et al. | 2 | 2 | 2 | 2 | 2 | 0 | 1 | 2 | 2 | 1 | 16 |
| Sui et al. | 2 | 2 | 2 | 2 | 2 | 0 | 1 | 1 | 2 | 2 | 16 |
